# Fyn kinase inhibition reduces protein aggregation, increases synapse density and improves memory in transgenic and traumatic Tauopathy

**DOI:** 10.1186/s40478-020-00976-9

**Published:** 2020-07-01

**Authors:** Si Jie Tang, Arman Fesharaki-Zadeh, Hideyuki Takahashi, Sarah Helena Nies, Levi M. Smith, Anin Luo, Annabel Chyung, Marius Chiasseu, Stephen M. Strittmatter

**Affiliations:** 1 Departments of Neurology and of Neuroscience, Program in Cellular Neuroscience, Neurodegeneration, Repair, Yale University School of Medicine, New Haven, CT 06536 USA; 2 Graduate School of Cellular and Molecular Neuroscience, University of Tübingen, D-72074 Tübingen, Germany; 3 Present address: Halda Therapeutics, 23 Business Park Drive, Branford, CT 06405 USA

**Keywords:** Tau, Fyn, Tauopathy, Alzheimer’s disease, Traumatic brain injury, Stress

## Abstract

Accumulation of misfolded phosphorylated Tau (Tauopathy) can be triggered by mutations or by trauma, and is associated with synapse loss, gliosis, neurodegeneration and memory deficits. Fyn kinase physically associates with Tau and regulates subcellular distribution. Here, we assessed whether pharmacological Fyn inhibition alters Tauopathy. In P301S transgenic mice, chronic Fyn inhibition prevented deficits in spatial memory and passive avoidance learning. The behavioral improvement was coupled with reduced accumulation of phospho-Tau in the hippocampus, with reductions in glial activation and with recovery of presynaptic markers. We extended this analysis to a trauma model in which very mild repetitive closed head injury was paired with chronic variable stress over 2 weeks to produce persistent memory deficits and Tau accumulation. In this model, Fyn inhibition beginning 24 h after the trauma ended rescued memory performance and reduced phospho-Tau accumulation. Thus, inhibition of Fyn kinase may have therapeutic benefit in clinical Tauopathies.

## Introduction

The microtubule-associated protein Tau (MAPT) accumulates in the brain of numerous neurological conditions, including Alzheimer’s disease (AD), Fronto-temporal Dementia, Progressive Supranuclear Palsy, and Chronic Traumatic Encephalopathy (CTE). The accumulated protein is hyperphosphorylated at multiple sites and misfolds to create paired helical filaments in neurofibrillary tangles. This accumulation is accompanied by synapse loss, gliosis, neurodegeneration and deficits of neurological function, including learning and memory and locomotion. Rare genetic mutations of *MAPT* itself demonstrate the causative role for this protein in neurodegeneration [46]. Furthermore, reduction of Tau expression is protective in several neurodegenerative models [12, 49, 70]. In recent years, Tau pathology has been recognized as a key feature of chronic late developing dementia after repetitive mild head trauma, in the syndrome of CTE [14, 37].

Amongst Tau-interacting proteins is the neuronally-enriched cytoplasmic tyrosine kinase, Fyn, a member of the Src family. Fyn physically associates with Tau, and has been reported to phosphorylate Tau at tyrosine near the N-terminus [3, 4, 32, 33]. Dendritic Tau is required to deliver Fyn to the post-synaptic density [24]. Fyn and Tau interact to modulate synapse density, behavior and electrophysiology in AD models [6, 7, 30, 48, 64]. Fyn is also implicated in AD pathogenesis by transducing signals downstream of Amyloid-ß oligomer binding to PrP^C^/mGluR5 receptor complexes at the cell surface [29, 30, 52, 63–65] and by interaction with the GWAS risk gene, PTK2B (Pyk2) [20, 27, 34, 53].

In order to limit AD pathophysiology, we have explored the possibility of inhibiting Fyn activity by repurposing Src family kinase inhibitors developed for oncology indications [27, 38, 58]. In preclinical models of Amyloid-ß-triggered deficits, Fyn inhibition with the orally available kinase inhibitor AZD0530 (saracatinib [22]) rescued synaptic loss, memory deficits and Tau accumulation [27, 56]. This approach has advanced to Phase 1b and Phase 2a clinical trials [39, 66]. Dose levels in AD subjects were limited by adverse events, and there was no improvement on primary outcome measures. Secondary imaging analyses showed non-significant trends for slowing the reduction in hippocampal volume and entorhinal thickness. Because the interaction of Fyn and Tau is direct, it remains possible that efficacy may be detected in a pure Tauopathy.

Here, we consider the role of Fyn inhibition in Tau-selective neurodegeneration using genetic and traumatic mouse models. Chronic inhibition of Fyn kinase activity in transgenic P301S Tau mice prevented neuronal phospho-Tau accumulation, microglial activation and pre-synaptic marker loss. Memory function was preserved by Fyn inhibition. The traumatic model combined low-grade repeated closed head injury with chronic variable stress to produce persistent memory dysfunction. Inhibition of Fyn kinase beginning 1 day after a 2-week-long injury period, reduced memory deficits and phospho-Tau accumulation. Fyn kinase inhibition may limit pathophysiology and reduce clinical symptoms derived from Tauopathy.

## Materials and methods

### Animals

For transgenic mice studies, B6;C3-Tg (Prnp-MAPT*P301S) PS19Vle/J (RRID:IMSR_JAX:008169) and B6C3F1/J (RRID:IMSR_JAX:100010) were purchased in April 2016 from Jackson Laboratories (JAX) and bred at Yale to obtain littermates of wild-types (WT) and transgenics (PS19) [71]. PS19 mice express a mutant human MAPT gene which results in a five-fold greater amount of human Tau proteins than the endogenous Tau produced naturally by mice. The PS19 mice were maintained in the hemizygous state, and a cohort of PS19 and WT littermates were randomly assigned to one of four experimental groups: WT, Vehicle; WT, AZD0530; PS19, Vehicle; PS19, AZD0530. Mouse genotyping was performed with a standard PCR assay as described on JAX website. DNA was extracted from ear tissue with REDExtract-N-Amp Tissue PCR kit (Sigma, XNAT) according to the manufacture’s protocol. There were two cohorts, each with the four groups, generated for these experiments (Supplementary Table S1). Average DOBs of the first and second cohorts are October 2016 and December 2018, respectively. Mice in the two cohorts were provided with chow formulated with either Vehicle or AZD0530 (depending on the experimental group) at 2 months of age and allowed to eat ad libitum until they were sacrificed at 9 months old and 11 months old, respectively*.* Similar results were obtained from the two cohorts in our behavioral tests.

For repetitive mild traumatic brain injury (rmTBI) plus stress studies, C57BL/6 J mice (RRID:IMSR_JAX: 000664) were purchased from JAX and bred for several generations at Yale. Only male mice were used for the combined rmTBI/stress model due to variation in chronic stress responses across the estrous cycle [26, 43], and to modulation of mouse TBI outcomes by sex [25, 57]. Since mice in the rmTBI/Stress study were dosed with AZD0530 by oral gavage, the mice were provided standard chow ad libitum*.* There were two cohorts with different treatment schedules for these experiments (Supplementary Table S1).

All protocols were approved by Yale Institutional Animal Care and Use Committee (IACUC). All animals were housed in groups with 2–4 animals per cage with access to food and water ad libitum*.* The housing light schedule had with a light period from 7 am to 7 pm and a dark period for the remaining 12 h.

### Chronic Oral dose preparation of AZD0530

AZD0530 (saracatinib) was prepared as described [27]. To generate chow containing AZD0530 for chronic dosing in PS19 experiments, the compound was incorporated into purified diet pellets by Research Diets, Inc. by dissolving the compound in a solution of 0.5% w/v Hydroxypropylmethylcellulose/ 0.1% w/v polysorbate 80 at 1.429 mg/ml. Vehicle pellets were purified diet pellets with control Vehicle solution (without drug). The dosage of the drug in the food was calculated to take into account the average amount of food eaten by a mouse in a single day per kg of weight [2] and adjusted to be equivalent to ingesting 5 mg/kg per day. Throughout the treatment period, the body weights of mice were monitored to ensure drug/food intake.

### Brain tissue collection

Mice were euthanized with CO_2_ and perfused with ice-cold PBS for one and a half minutes. The brains were dissected and the hemispheres were divided. The hippocampus and cortex were dissected from the left hemisphere and were individually snap frozen in liquid nitrogen to be used for biochemical analysis. The right hemispheres were fixed in 4% paraformaldehyde in PBS for 24 h at 4 °C and then placed in PBS with 0.05% Azide to be used for immunohistochemistry.

### Mouse brain protein extraction

Mouse brain protein extraction was performed as previously described [60] with modifications. The hippocampi were weighed and then homogenized with 20 strokes in ten-fold volume (w/v) of ice-cold 50 mM Tris-HCl, pH 7.5, 150 mM NaCl, PhosSTOP, cOmplete-mini protease inhibitor cocktail (Roche), and 1 mM vanadate. After ultracentrifugation for 20 min at 100,000 x g at 4 °C, the supernatants were collected as TBS-soluble fractions, and the TBS-insoluble pellet was re-suspended in RIPA (25 mM Tris-HCl pH 7.5, 150 mM NaCl, 1% NP40, 0.5% sodium deoxycholate, 0.1% SDS, PhosSTOP, cOmplete-mini (Roche), and 1 mM vanadate) at a volume equivalent to the amount used in the TBS extraction. The samples were incubated in RIPA for 30 min at 4 °C and then ultracentrifuged for 20 min at 100,000 x g at 4 °C. The supernatants were collected as RIPA-soluble fractions.

### Immunohistochemistry

Immunohistochemistry was performed as previously described [27] with slight modifications. Forty μm coronal sections of the right hemisphere were cut with a Leica VT1000S Vibratome. Antigen retrieval was performed on the forty μm free-floating sections by incubating three slices from each mouse in 1x Reveal decloaker buffer (Biocare Medical) in 24-well-plates for 10 min at 90 °C in an oven and then cooled down at room temperature for 10 min. The antigen retrieval step was done for PHF1, AT8, HT7, and GFAP stainings. Sections were permeabilized with 0.1% Triton X-100 at room temperature for 5 min for PHF1, HT7, and SV2A staining and for 30 min for CD68/Iba1, AT8, and GFAP. All sections were blocked with 10% donkey, horse, or goat serum in PBS for 1 h at room temperature. The sections were then incubated in primary antibody in 4% donkey, horse, or goat serum in PBS overnight at room temperature. For SV2A, HT7, and PHF1 stainings, primary antibodies were incubated at 4 °C rather than room temperature. The primary antibodies that were used include: PHF1 (gift from Dr. Peter Davies, Albert Einstein College of Medicine, Bronx, NY 1:250), SV2A (Abcam, Ab32942, 1:500), CD68 (Biorad, MCA1957, 1:900), Iba1 (Wako, 019–19,741, 1:500), AT8 (Invitrogen, MN1020, 1:500), GFAP (Abcam, Ab7260, 1:1000), pTyr18 (Medimabs, MM-0194-P, 1: 200), Tau (DAKO, A0024,1:5000), HT7 (Invitrogen, MN1000, 1:500), and NeuN (Millipore, ABN91, 1:500). The sections were then washed three times with PBS for 5 min each and then incubated for 1–2 h at room temperature in either donkey anti-rabbit or donkey anti-mouse fluorescent secondary antibodies in PBS (Invitrogen Alexa Fluor 1:500). After incubation, the sections were washed three times with PBS for 5 min. To quench autofluorescence for PHF1, AT8, and GFAP stainings, sections were dipped briefly in dH_2_O and then incubated in copper sulfate solution (10 mM copper sulfate, 50 mM ammonium acetate, pH 5) for 15 min before dipping back into dH_2_O and then placed in PBS [55]. All sections were mounted onto glass slides (Superfrost, Fischer Scientific Company L.L.C.) and coverslipped with Vectashield (Vector) antifade mounting medium with DAPI.

### Immunoblot

Immunoblotting was performed as previously described [16] with modifications. In general, the RIPA-soluble fraction was mixed in 2x Laemmli Sample Buffer (Bio-Rad) with 0.5% β−mercaptoethanol. For Tau extracted from human brains, samples were diluted with 1x Laemmli Sample Buffer (containing no β−mercaptoethanol) to 10, 5% or 2.5% of their initial concentration to evaluate Tau concentration. The mixture was heated for 5 min at 95 °C and then loaded into precast 4–20% Tris-glycine gels (Bio-Rad) to be electrophoresed. The protein was then transferred with an iBlot 2 Transfer Device onto nitrocellulose membranes (Invitrogen IB23001) and then incubated in blocking buffer (Rockland) for 1 h at room temperature. Membranes were then incubated overnight at 4 °C in blocking buffer with primary antibodies: Fyn (Cell Signaling, 4023, 1:1000), pSRC (Tyr416) (Cell Signaling, 6943, 1:1000), β-actin (Cell Signaling, 3700, 1:2000), total Tau (HT7) (Invitrogen, MN1000, 1:1000) and p-Tau (Invitrogen, AT180, 1:1000). The next day, membranes were washed three times with TBST for 5 min and incubated in secondary antibodies (donkey anti-rabbit (800) and donkey anti-mouse (680), Li-Cor IR Dye) for 1 h at room temperature. Membranes were washed three times with TBST for 5 min, visualized with an Odyssey Infrared imaging system (Li-Cor), and then the immunoreactive bands were quantified with ImageJ software.

### Tau extraction from human brains

Pre-existing de-identified human autopsy brains were accessed for these studies under conditions considered exempt from Human Subjects regulation after review of the Institutional Review Board at Yale. Fresh frozen brain had been stored at − 80 C. The AD brain used in this study derived from a male, age 87, 23 h post-mortem interval, National Institute on Aging classification: A2, B3, C2 [23]. The neurologically intact control brain had no signs or minimal signs of AD-associated histopathology, with Braak stage 0-II and CERAD neuritic plaque score of “none” or “sparse”. Tau was extracted based on a previously published protocol [19] with some modifications. Briefly, 11–12 g of cortical grey matter were dounce homogenized in 30 mL lysis buffer [10 mM Tris-HCl, 1 mM EDTA, 0.1% sarkosyl, 10% sucrose, freshly added 2 mM DTT, phosSTOP (Roche) and protease inhibitors (Roche)]. Throughout the extraction, lysates were kept on ice. Homogenates were centrifuged at 12,000 rpm at 4 °C for 12 min (Ti 45 rotor, Beckman Coulter). The supernatant was pooled, and the pellets were re-extracted and centrifuged twice more as above. The pooled supernatant was centrifuged again twice at 12,000 rpm at 4 °C for 12 min (Ti 45 rotor, Beckman) to remove debris. Then, the sarkosyl concentration was increased to 1% and samples were nutated for 1 h at room temperature (RT). The samples were centrifuged at 300,000 x g for 1 h at 4 °C (57,000 rpm, Ti 70 rotor, Beckman Coulter). The resulting pellet was washed with PBS supplemented with phosSTOP and protease inhibitors twice and then resuspended in PBS supplemented with phosSTOP and protease inhibitors. After sonication at 15% amplitude for 20 s with 0.5 s ON/0.5 s OFF pattern, the lysate was centrifuged at 100,000 x g for 30 min at 4 °C. The supernatant was discarded, and the pellet washed twice in PBS supplemented with phosSTOP and protease inhibitors. The pellet was once more resuspended in PBS supplemented with phosSTOP and protease inhibitors and sonicated at 30% amplitude for 60 s with 0.5 s ON/0.5 s OFF pattern. This was followed by a 100,000 x g spin for 30 min at 4 °C. The resulting supernatant contained the soluble Tau and was aliquoted and frozen at − 80 °C until further analysis or experimental use. The concentration of Tau in the extract was ~ 0.3 μg/μL by western blot analysis using recombinant human Tau.

### In vitro tau seeding activity

Primary mouse neuronal culture was prepared as described [28]. Pregnant mice were euthanized with CO_2_. Hippocampal and cortical tissues (1:1 ratio) were harvested from E17 embryos (both male and female) on ice cold Hibernate E media (BrainBits, HE) and digested in 0.05% Trypsin (Gibco), and 1 mg/mL DNase (Sigma DN25) in HBSS for 10 min at 37 °C. After incubation, neurons were triturated manually in Neurobasal-A media (Gibco) supplemented with B27, 1 mM sodium pyruvate, GlutaMAX, and 100 U/mL penicillin and 100 μL streptomycin (all from Gibco) at 37 °C. Dissociated neurons were spun at 250 x g at 4 °C for 6 min. Neurons were plated at 75,000 cells/well onto PDL-coated 96-well plates (Corning #354461) in the same Neurobasal-A media with supplements.

In vitro Tau seeding experiments were performed as previously described [19] with modifications. One week after primary neurons were plated onto PDL-coated 96-well plates (DIV7), Tau extracts (~ 150 ng of Tau /well) from human AD brains were seeded into wells. Neurons were also treated with 0.5 or 1 μM AZD0530 in high purity water. At DIV21, neurons were fixed with ice cold methanol for 30 min on ice and blocked with 10% normal donkey serum and 0.2% Triton X-100 in PBS for 30 min. Then, neurons were incubated with primary antibodies diluted in 1% normal donkey serum and 0.2% Triton X-100 in PBS overnight at 4 °C: Anti-MAP 2 (Cell Signaling, 4542, 1:150) and mouse Tau (T49) (Millipore sigma, MABN827, 1:500). The samples were washed three times with PBS and incubated in secondary antibodies (Invitrogen Alexa Fluor 1:500) diluted in 1% normal donkey serum and 0.2% Triton X-100 in PBS for 1 h and DAPI.

### HEK-293 Proximity Ligation Assay (PLA)

HEK-293 T cells were maintained in Dulbecco’s Modified Eagle Medium (DMEM) supplemented with 10% fetal bovine serum (FBS) and 1% penicillin/streptomycin (100 U/mL). Cells were plated at 40,000 cells/well onto 8-well chamber slides (Thermo Scientific 154,941). Transient transfection with plasmids expressing human Tau (Origene, RC213312) and Fyn (Origene, RC224691) was performed using Lipofectamine 2000 transfection reagent (Invitrogen). Three hours later, 2 μM AZD0530 in DMSO or DMSO (vehicle) was added to the treatment or control wells. Twenty-four hours after treatment, cells were fixed in 4% paraformaldehyde in PBS at room temperature for 30 min and then washed 3X in PBS for 5 min and stored until PLA was performed.

Duolink In Situ Detection Reagents Green (Sigma DUO92014) were used for the PLA as described [51] with modifications. HEK-293 T cells were fixed on 8-well Chamber Slides and permeabilized/blocked with 10% normal donkey serum, 0.2% Triton X-100 in PBS for 30 min at room temperature. Wells were then incubated with primary antibodies Tau (DAKO, A0024, 1:4000) and Fyn15 (Santa Cruz, sc-434, 1:500) in 1% normal donkey serum in PBS overnight at 4 °C. The next day, after removing wells, the slides were washed 3x for 5 min in PBS and then incubated for 1 h at 37 °C in 8 μL Duolink In Situ PLA Prole Anti-Rabbit PLUS (Sigma DUO92002) and 8 μL Duolink In Situ PLA Probe Anti-Mouse MINUS (Sigma DUO92004) in 24 μL 1% normal donkey serum in PBS per sample. Slides were then washed 2X for 5 min with 1x Wash Buffer A (Sigma DUO82049) at room temperature. For the ligation step, slides were incubated for 1 h at 37 °C in 8 μL 5X Ligation buffer and 1 μL of Ligase in 32 μL high purity water. Then, slides were washed 2x for 5 min in 1x Wash Buffer A at room temperature. For the amplification step, the slides were incubated for 100 min at 37 °C in 8 μL 5x Amplification buffer and 0.5 μL polymerase in 31.5 μL high purity water per sample. The slides were then washed 2X for 10 min in 1x Wash Buffer B (Sigma DUO82049) and for 1 min in 0.01x Wash Buffer B and then 5 min in PBS at room temperature. For Tau and Fyn visualization, slides were incubated for 1 h at room temperature in the secondary antibodies donkey anti-rabbit conjugated with Alexa Fluor-568 and donkey anti-mouse conjugated with Alexa Fluor-647 (ThermoFisher 1:500) with 1% normal donkey serum in PBS. Then, slides were washed 4X for 5 min in PBS. Coverslips were mounted on the slides with Vectashield (Vector) antifade mounting medium with DAPI and stored at 4 °C until imaging.

### Imaging and analysis

For imaging of sections from immunohistochemistry, Nikon Eclipse Ti Spinning Disc Confocal Microscope was used with a 40X 1.3 NA oil-immersion lens in Cargille immersion oil. A Zeiss AxioImager Z1 fluorescent microscope was used with a 5X objective. The dentate gyrus, CA1, and CA3 of mice were imaged and the percent positive area for each staining was analyzed with a macro in ImageJ or a pipeline in CellProfiler. For Iba1/CD68 images, z-stacks of the images were compressed into a maximum intensity projection with Volocity software before analyzing with CellProfiler. Iba1-positive area were identified with CellProfiler and masked over corresponding CD68 image. The percent of CD68-positive area within the Iba1 mask was calculated. Aperio ImageScope software was used for cresyl violet images.

For imaging of PLA, images were taken with Zeiss 800 confocal microscope was used with 20X 0.8 air-objective lens or Leica DMi8 with 20X 0.75 air-objective lens. Four pictures were taken per condition in each experiment. Z-stacks were compressed into a maximum intensity projection with ZEN software before analyzing with ImageJ. The area covered by Tau fluorescence was measured and masked over the corresponding PLA and Fyn image. The percent area of Fyn-Tau PLA-positive area within Tau-positive area and the percent of Fyn-positive area within Tau-positive area was calculated. The values were normalized to that of non-treated sample.

For the in vitro Tau seeding activity analysis, images were automatically taken using ImageXpress Micro XLS (Molecular Devices) (20X objective lens). Each experiment was performed in triplicate and four images were taken per well. With ImageJ, MAP 2-positive area was identified and masked over the corresponding T49 image. The percent of T49-positive area within the MAP 2 mask was calculated.

All images analyzed on ImageJ were uniformly thresholded for area analysis. All the imaging and analyses were conducted by a researcher who was blinded to the genotype and treatment type.

### Morris water maze

The Morris water maze (MWM) paradigm was performed as previously described [27]. When conducting all behavioral tests, the investigator was blinded to the mouse’s genotype and pharmacological treatment. Prior to behavioral tests, each mouse was handled for 5 min for 4 days to reduce anxiety. Mice were placed in a pool with a hidden, clear platform filled with water to 1 cm above the submerged platform. The hidden platform was placed in one of the four quadrants of the pool with the 4 drop zones directly across from the platform. At each of the four cardinal directions, a symbol, such as a plus or a cross, was placed as possible recognition flags. At the beginning of each day, mice were habituated in the behavior room for an hour before MWM began. For three consecutive days, two times each day, mice were dropped off facing the wall at four different drop zones (four trials between 9 am – 2 pm and four between 3 pm – 8 pm). Each trial was performed by alternating two mice (A1, A2, A1, A2, A1, A2 … etc). Latency was measured as the time that it took for the mouse to find and spend 1 s on the hidden platform. If there was a failure to reach the platform in 60 s, the mouse was guided to the platform and allowed to rest on it for 15 s. On the fourth day, a probe trial was performed, in which the platform was removed and mice was allowed to swim in the pool for 60 s.

In the subsequent trials (reverse learning and probe trials), the order in which the mice were placed in the pool was reversed, and the swim procedure was repeated with the hidden platform relocated diagonally from the initial platform location, and the drop zones were also altered to be directly diagonal from the forward swim drop zones.

After reverse learning and probe trials, a flag was placed atop of the hidden platform and mice were repeatedly placed in the pool. Time taken to reach the visible platform was recorded. When a consistent time for a mouse was reached, the last three times were averaged and the overall average of latency to hidden platform was used to exclude mice that were outliers from analysis due to visual impairments. Latencies and distance traveled for all trials were measured with the Panlab SMART Video Tracking Software.

### Passive avoidance test

A Passive Avoidance Controller CAT 7551 was used to conduct the passive avoidance test as previously described [21] with slight modifications. The door delay was set to 90 s, and the shock intensity was set to 0.5 mA with a shock duration of 2 s. A mouse was placed into the white box with a light source overhead and given 5 min to cross through the door and into the opaque, black box after 90 s of acquisition in the white box. In trial 1, the mouse received a foot shock once it passed through to the black box. Trial 1 began at 10 am, after an hour of habituation in the behavior room. For trial 2, the mouse was placed back in the white box approximately 5 min after trial 1 and was shocked if it passed through to the black box. Twenty-four hours after trial 1, trial 3 was conducted with the shock intensity lowered to 0.0 mA. Mice were excluded if they failed to cross into the black box after 5 min during trial 1. Experimenter was blinded to the genotype of the mouse.

### Rotarod performance test

The Rotarod test was performed as previously described [72]. Mice were habituated in the behavior room for an hour before Rotarod test began at approximately 1 pm. Mice were placed atop a Rotarod (Economex Columbus Instruments) that was set to accelerate at 0.3 rpm/s until 4 rpm. Five trials were performed on each mouse with two-minute rests in between each trial. The time that each mouse stayed on the rod was recorded. Experimenter was blinded to the genotype of the mouse.

### Repetitive Mild Traumatic Brain Injury (rmTBI) plus stress studies

The injury combined Closed Head Injury (CHI) and Chronic Variable Stress (CVS). Control mice received Sham-CHI and Sham-CVS treatments. There is a wide range of rodent head injury models [36, 47, 69]. These include lateral fluid percussion [62], controlled cortical impact [50], weight drop [13] and blast injury [17]. The rmTBI model used here is CHI [54]. This model offers multiple advantages, which include the capacity to titrate the intensity of impact, depth of injury and duration of impact. Most critically, it does not involve craniotomy, thereby facilitating multiple mild injuries and reducing infectious complications [5].

Multiple preclinical studies have explored the interaction of TBI with psychiatric disease [9, 41, 44, 45, 61]. Combined stress and TBI increased neuroinflammation, axonal injury and behavioral deficits [40]. CVS induces Tau phosphorylation at Ser396 and Ser404 [68]. Unpublished work by one of us (A.F.Z.) showed that stress preceding injury generated more severe behavioral deficits and greater neuroinflammation. The injury/stress model used in this study is based on these studies, and employed 14 days of CVS proceeding mild CHI on each day, with the side of the CHI alternating between days.

#### Chronic Variable Stress (CVS)

Stress was induced as described [42]. The CVS was comprised of exposure to 5 different aversive stimuli over a 14 day period. The stimuli included: 3-min cold water swim at 16–18 °C, overnight food deprivation with access to water ad libitum, 3 h in a cage with 300 ml of water added, 3 h exposure to a cage tilted at 45 degrees, and 15 min immobilization in a flat-bottomed restraint chamber (Braintree Scientific Instruments). The mice were exposed to a set of three pre-randomized aversive stimuli on each given day during the 14 day period, in order to simulate the unpredictable nature of psychological trauma, while limiting habituation. Sham-CVS were transferred to single-housed cages for the same time period but not otherwise stressed. The CVS was conducted consistently between 8 am and 1 pm.

#### Closed Head Injury (CHI)

Within 1–3 h of CVS exposure on each of 14 consecutive days, isoflurane-based anesthesia was induced for 3 min with isoflurane (3.5% in oxygen (1.0 L/min) and maintained (3% in oxygen (1.0 L/min) until immediately after the impact. The head of the mouse was shaved and CHI was induced using a 5.0 mm diameter tip operated by an electromagnetic impactor (Leica Microsystems, Buffalo Grove, IL). The 5 mm diameter impactor tip was placed 5 mm lateral from the sagittal line, 5 mm caudal from the eye, at an angle of 20° from the vertical with an impact velocity 5 m/sec, impact depth of 1 mm and 100 msec dwell time. Sham-CHI mice were shaved and anesthetized in the same manner, but did not undergo impaction. The total anesthesia exposure during each procedure did not exceed 6 min for any mice, and no hypothermia was detected in this short period. The mice core body temperature was closely controlled and monitored using a heat pad (36.5–37.5 C), and a rectal probe. Mice were placed on a heating pad to maintain body temperature while receiving the impact, as well as during the post-injury recovery period. The mice underwent a total of 14 consecutive days of CHI injury, once per day. The site of injury alternated between right and left hemispheres on consecutive days to produce diffuse injury. By alternating sides on different days, the surgical procedure was substantially simplified and anesthesia time was kept to a minimum. Injury severity was assessed using time interval between injury and recovery of the righting reflex [18]. Mice were returned to standard vivarium after restoration of their righting reflex, typically less than 5 min after CHI. The CHI was conducted between 3 pm and 7 pm.

#### Schedule for rmTBI/stress treatment with AZD0530

In the first treatment experiment, mice began treatment on Day 15 (24 h following the last injury on Day 14) with AZD0530 at 5 mg/kg/day in two equally divided doses by oral gavage for a 10 week period as described [72]. The Vehicle for the drug was 0.5% wt/vol hydroxypropyl-methylcellulose (HPMC)/0.1% wt/vol polysorbate 80, and each dosing volume was approximately 250 μl [27].

In the second treatment experiment, the mice underwent a 10 week treatment starting on Day 121, or 107 days after the last day of injury.

Upon completion of the treatment period, both groups underwent a weeklong period of behavioral assays including Morris Water Maze (MWM), and novel object recognition, as described [27]. Each mouse was handled for 5 min for the 5 days preceding the behavioral testing. The mice received the continued oral gavage treatment during the testing period.

#### Cresyl violet staining of rmTBI/stress brain

To assess tissue damage, coronal sections were stained with cresyl violet (Sigma Aldrich, C5042) for 10 min, washed in water for 3 min, de-stained in 95% ethanol for 10 min, and then dehydrated with 100% ethanol for 5 min twice and xylene for 5 min twice. Sections were then mounted with CytoSeal60 (ThermoFischer, 8310–4).

### Quantification and statistical analysis

One-way ANOVA with Dunnett’s multiple comparisons test, two-way ANOVA with Sidak’s multiple comparisons test, t-test, or Wilcoxon match-pairs signed tests were performed as specified in the figure legends using GraphPad Prism 8. All n-values represent individual mice. For IHC, each data point represents the average of three brain sections from one animal. For behavioral tests, the number of trials that each data point represents can be found in the figure legends. Values are represented as mean ± SEM. Statistical significance is determined if *p* < 0.05.

## Results

### Fyn inhibition rescues behavioral deficits of tau transgenic mice

**Fig. 1:**
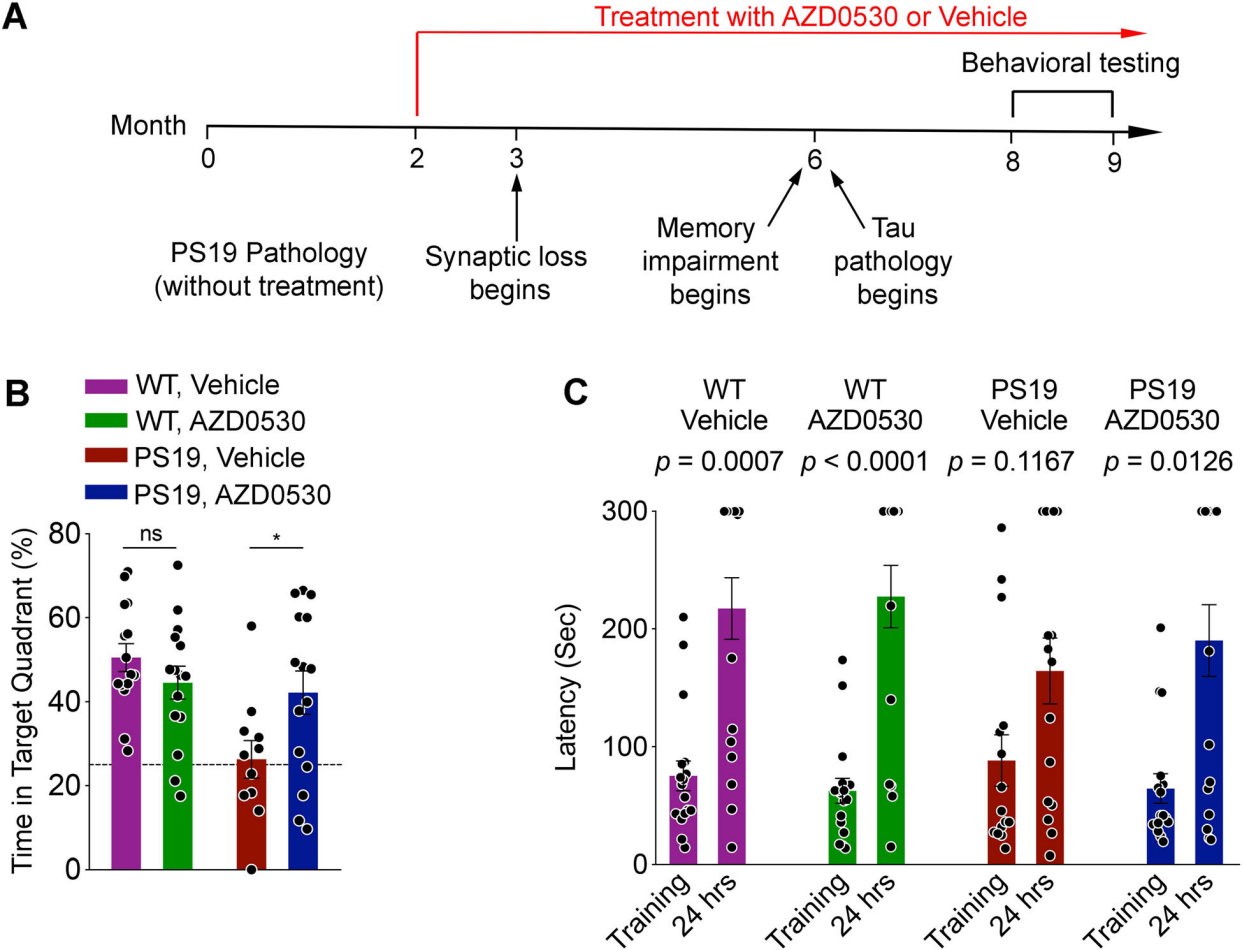
Fyn Kinase Inhibition Prevents Memory Deficits in P301S Tau Transgenic Mice. **(a)** Timeline of PS19 mouse treatment. **(b)** Morris water maze probe trial for 8-month-old PS19 or WT mice after 6 months of AZD0530 or Vehicle treatment. Twenty four hours after the reverse learning trials in the Morris water maze, the submerged platform was removed and the fraction of 60 s spent in the target quadrant where the hidden platform had been located previously was recorded. *n* = 11–15 /group, each dot is one mouse. Data are mean ± SEM. Dashed line indicates random chance performance. Two-way ANOVA reveal an interaction between genotype and treatment (*p* = 0.014). *p < 0.05; Sidak’s post-hoc multiple comparisons test. **(c)** Passive avoidance test for 8-month-old PS19 and WT mice after 6 months of treatment with AZD0530 or Vehicle. Latency was measured as the time for the mouse to cross to the opaque box. *n* = 16–18 /group. Wilcoxon match-pairs signed test; (WT, Vehicle: *p* = 0.0007; WT, AZD0530: *p* < 0.0001; PS19, AZD0530: *p* = 0.1167; PS19, AZD0530: *p* = 0.0126)

The PS19 transgenic mouse strain is a commonly used mouse model of Tauopathy that expresses human 1N4R Tau with frontotemporal dementia-associated P301S mutation [71]. This PS19 model exhibits Tau pathology and recapitulates several phenotypes observed in human Tauopathies. Three-month-old PS19 mice begin to exhibit synaptic loss in the hippocampus, and at 6 months of age, cognitive impairments and Tau pathology have been observed [71]. Thus, to test prophylactic effects of Fyn inhibition in the PS19 mice, cohorts of PS19 and WT mice were treated with Vehicle or AZD0530 starting at 2 months of age, prior to any Tau-associated pathology progression and treated chronically (Fig. 1a).

To achieve chronic dosing of a Fyn kinase inhibition, mice were fed a diet of food pellets supplemented with AZD0530 at a dose calculated to achieve 5 mg/kg/d of active compound based on reported average consumption [2]. We assessed the effectiveness of AZD0530 supplemented in the purified diet pellets in the brain using WT mice treated with AZD0530 or Vehicle for 9 months. Immunoblot analysis with phospho-Src (pY416) antibody using the RIPA-soluble fraction of hippocampus revealed a significant reduction in phosphorylation of Y416, a marker for activation of Src-family tyrosine kinase, in the hippocampus of AZD0530-treated WT mice compared to that of Vehicle-treated WT mice (Supplementary Fig. S1A, S1B). There was no difference in total Fyn levels between WT mice with and without AZD0530 treatment (Supplementary Fig. S1A, S1C). Thus, AZD0530 formulated in diet pellets crosses the blood brain barrier and inhibits Fyn in the mouse brain. Coupled with previous pharmacokinetic data demonstrating the presence of AZD0530 in the brain and cerebrospinal fluid of treated mice [27, 39], these findings demonstrate that chronic AZD0530 administration in chow achieves drug levels sufficient to achieve sustained Fyn inhibition.

We assessed spatial learning and memory of Vehicle and AZD-treated WT and PS19 mice at 8 months of age using the Morris water maze. In the forward and reverse learning trials, no statistically significant learning deficits were observed in the Vehicle-treated PS19 mice although the Vehicle-treated PS19 mice showed a trend towards increased escape latency the hidden platform in the reverse learning trial compared to AZD0530-treated PS19 mice and both WT groups (Supplementary Fig. S2A, S2B). In the probe trial following these reverse learning trials, the Vehicle-treated PS19 mice spent significantly less time in the target quadrant than their WT littermates, reflecting a spatial memory deficit (Fig. 1b). Notably, AZD0530 treatment significantly improved this memory deficit in PS19 mice (Fig. 1b). Although a slight increase in the time to reach the platform was observed in Vehicle-treated PS19 mice in the visible platform trial, we did not observe a motor impairment in 8-month-old PS19 mice in the Rotarod test (Supplementary Fig. S2C and S2D).

We also tested fear-associated learning for the same cohorts of mice using the passive avoidance test, where mice were placed inside a light-filled box with a door to a dark box. In this paradigm, mice that enter over to the dark box are given a mild foot shock and learning is scores as delayed entry into the dark box on subsequent trials. While both groups of WT mice and the AZD0530-treated PS19 mice exhibited passive avoidance at 24 h after association of the foot shock with the dark box, the Vehicle-treated PS19 mice did not learn to associate the dark box with the foot shock (Fig. 1c). Together, these data demonstrate that chronic AZD0530 treatment reduces cognitive impairments in PS19 mice at 8 months of age.

### Accumulation of phospho-tau is reduced by AZD0530

**Fig. 2:**
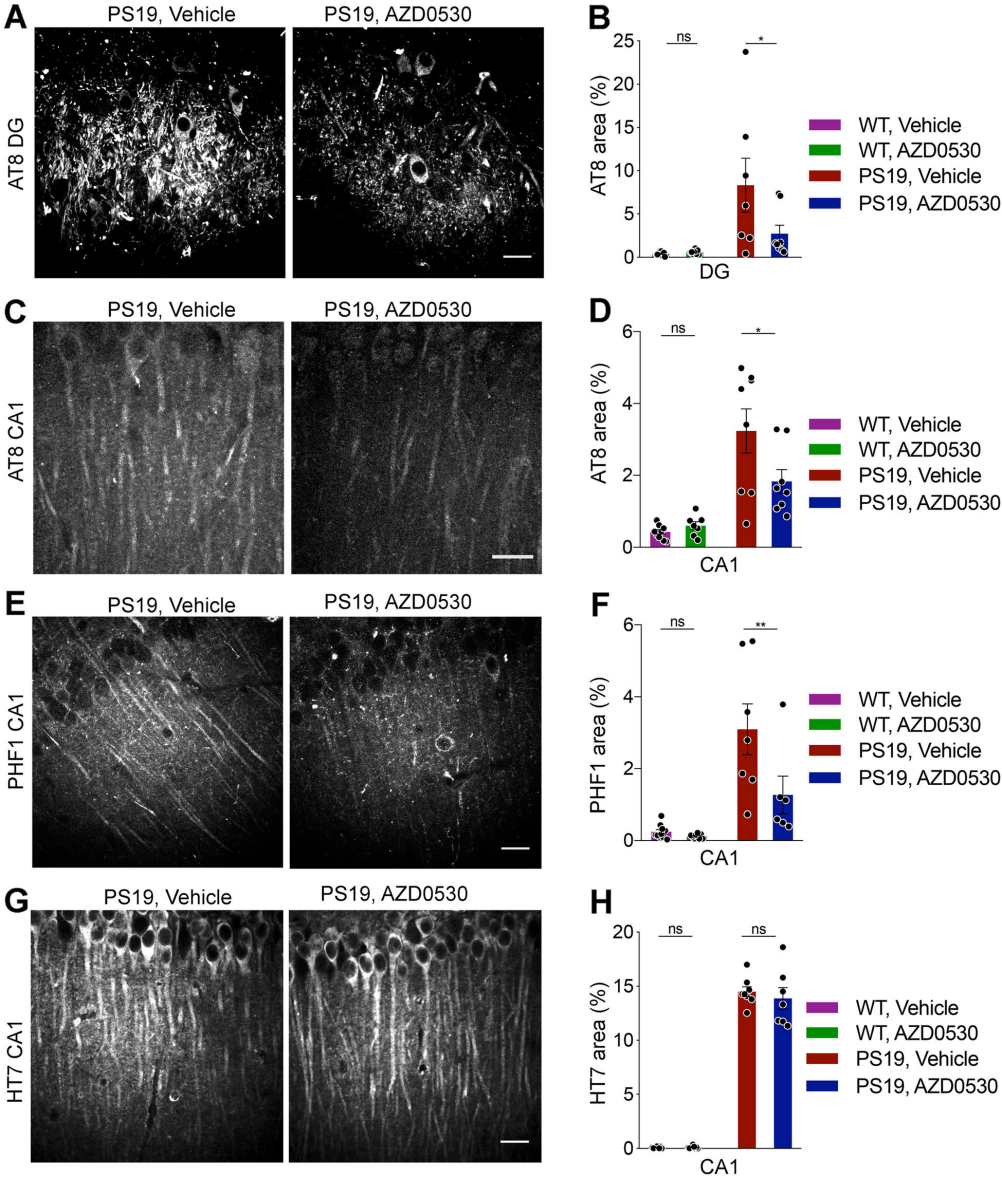
Reduced phospho-Tau Accumulation in Transgenic Mice Treated with AZD0530. **(a)** Representative images of AT8 immunoreactivity in the dentate gyrus (DG) of the hippocampus in 9-month-old PS19 mice after 7 months of treatment with AZD0530 or Vehicle. Scale bar, 20 μm. **(b)** Quantification of AT8-positive area (%) in the dentate gyrus of the hippocampus in 9-month-old PS19 and WT mice after 7 months of treatment. Data are mean ± SEM. *n* = 7–10 /group, each dot is the average of three sections from one mouse. Two-way ANOVA revealed an interaction between genotype and treatment (*p* = 0.0487). *p < 0.05; Sidak’s post hoc multiple comparisons test. **(c)** Representative images of immunofluorescent staining for AT8 in the CA1 of the hippocampus in 9-month-old PS19 mice after 7 months of treatment. Scale bar, 20 μm. **(d)** Quantification of AT8-positive area in the CA1 of the hippocampus in 9-month-old PS19 and WT mice after 7 months of treatment. Data are mean ± SEM. *n* = 7–10 /group, each dot is the average of three sections from one mouse. Two-way ANOVA revealed an interaction between genotype and treatment (*p* = 0.03). *p < 0.05; Sidak’s post hoc multiple comparisons test. **(e)** Representative images of immunofluorescent staining for PHF1 in the CA1 of the hippocampus in 9-month-old PS19 mice after 7 months of treatment. Scale bar, 20 μm. **(f)** Quantification of PHF1-positive area in the CA1 of the hippocampus in 9-month-old PS19 and WT mice after 7 months of treatment. Data are mean ± SEM. *n* = 6–10 /group, each dot is the average of three sections from one mouse. Two-way ANOVA revealed an interaction between genotype and treatment (*p* = 0.0341). **p < 0.01; Sidak’s post hoc multiple comparisons test. **g)** Representative images of immunofluorescent staining for HT7 in the CA1 of the hippocampus in 9-month-old PS19 mice after 7 months of treatment. Scale bar, 20 μm. **(h)** Quantification of HT7-positive area in the CA1 of the hippocampus in 9-month-old PS19 and WT mice after 7 months of treatment. Data are mean ± SEM. *n* = 7–9 /group, each dot is the average of three sections from one mouse. Two-way ANOVA revealed a main effect of genotype (*p* < 0.0001) but no interaction between genotype and treatment (*p* = 0.5181); Sidak’s post hoc multiple comparisons test

The most likely explanation for the observed improvements in cognitive function is reduced accumulation of transgene-dependent Tau as a result of Fyn inhibition. We examined whether AZD0530 treatment reduces Tau pathology in PS19 mice at 9 months of age. Immunohistochemistry using the AT8 antibody directed against phospho-Tau (Ser202/Thr205) showed a significant increase in AT8 immunoreactivity in the dentate gyrus (DG) and CA1 areas of hippocampus of PS19 mice compared to WT mice (Fig. 2a-d). Strikingly, chronic AZD0530 treatment significantly mitigates the increase in AT8 immunoreactivity in PS19 mice. Similarly, immunostaining using PHF1 antibody directed against phospho-Tau (Ser396/Ser404) revealed a significant decrease in PHF1-immunoreactive area in the CA1 area of the hippocampus of AZD0530-treated PS19 mice compared to Vehicle-treated PS19 mice (Fig. 2e, f). In contrast, AZD0530 treatment had no significant effects on the levels of total human Tau expressed from the transgene (Fig. 2g and h). We did not observe thioflavin-S-positive inclusions in the DG and C1A areas of any of these mice (data not shown). Therefore, the phosphorylated Tau had not matured neurofibrillary tangle-like pathology at this age. These results demonstrate that chronic inhibition of Fyn by AZD0530 treatment reduces phospho-Tau pathology without changing total human Tau levels in the hippocampus of PS19 mice.

### Tau transgene-induced gliosis is lessened by Fyn inhibition

**Fig. 3:**
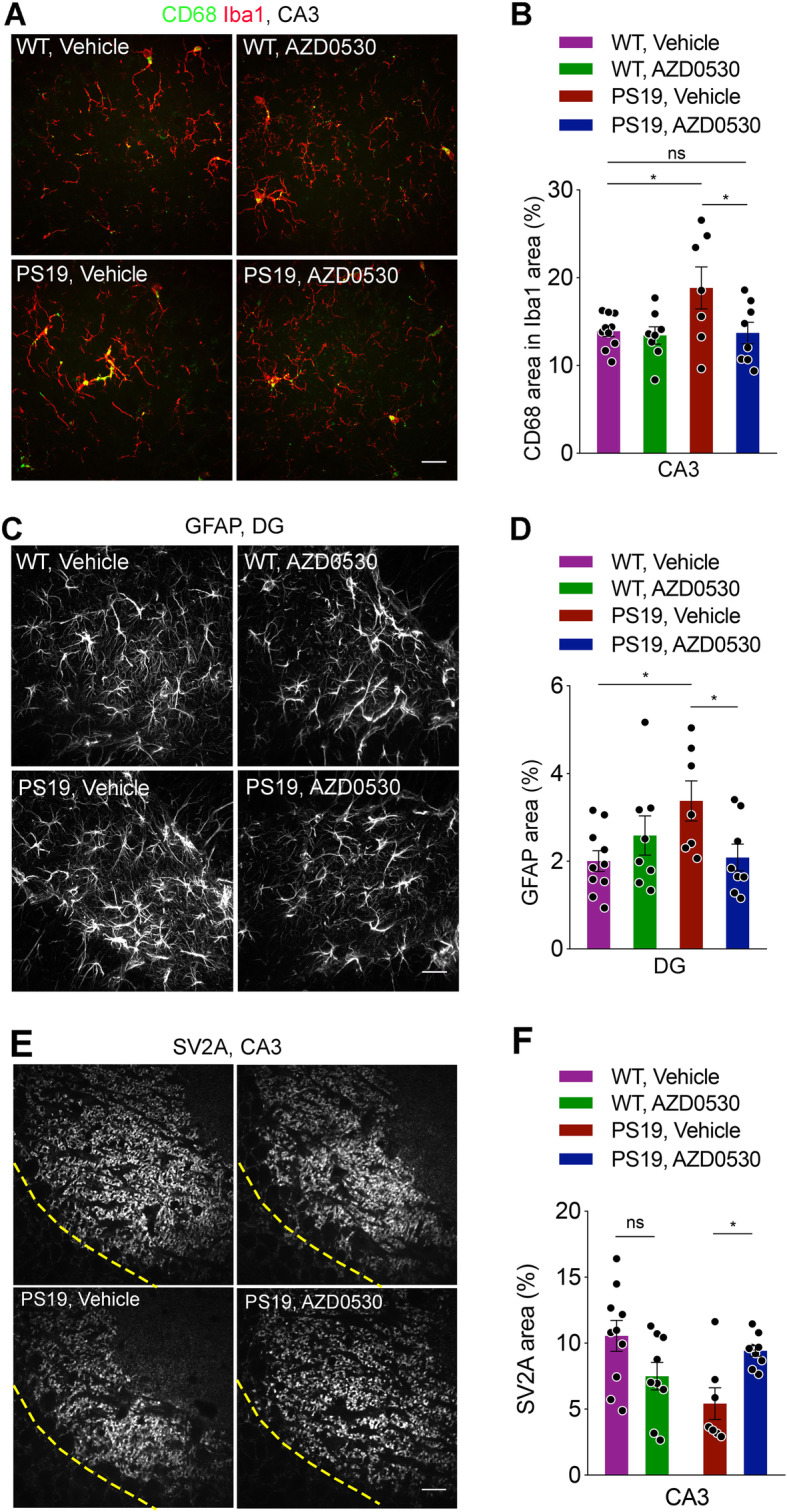
Chronic Fyn Inhibition Prevents Glial Activation and the Loss of Presynaptic Marker SV2A in Mutant Tau Transgenic Mice. **(a)** Representative images of CD68 and Iba1 double immunostaining in the CA3 region of the hippocampus from 9-month-old PS19 and WT mice after 7 months of treatment with AZD0530 or Vehicle. Scale bar, 20 μm. **(b)** Quantification of CD68-positive area (%) within Iba1-immuoreative area in the CA3 segment of the hippocampus from 9-month-old PS19 and WT mice after 7 months of treatment. Data are as mean ± SEM. *n* = 7–10 /group, each dot is the average of three sections from one mouse. *p < 0.05; One-way ANOVA with Dunnett’s multiple comparisons test. **(c)** Representative images of immunofluorescent staining GFAP in the dentate gyrus (DG) of the hippocampus in 9-month-old PS19 and WT mice after 7 months of treatment. Scale bar, 20 μm. **(d)** Quantification of GFAP-positive area (%) in the dentate gyrus of the hippocampus in 9-month-old PS19 and WT mice after 7 months of treatment. Data are mean ± SEM. *n* = 7–10 /group, each dot is the average of three sections from one mouse. *p < 0.05; One-way ANOVA with Dunnett’s multiple comparisons test. **(e)** Representative images of immunofluorescent staining for SV2A in the CA3 region of the hippocampus from 9-month-old PS19 and WT mice after 7 months of treatment with AZD0530 or Vehicle. Dashed lines represent the divide between the cell body layer and synaptic region. The cell bodies in the image were used to capture similar ROI from each section. Scale bar, 20 μm. **(f)** Quantification of SV2A-positive area (%) in the CA3 of the hippocampus in 9-month-old PS19 and WT mice after 7 months of treatment. Data are mean ± SEM. *n* = 7–10 /group, each dot is the average of three sections from one mouse. Two-way ANOVA revealed an interaction between genotype and treatment (*p* = 0.0015). *p < 0.05; Sidak’s post hoc multiple comparisons test

As previous studies have shown that pathological Tau induces neuroinflammation leading to synapse loss in PS19 mice [1, 11, 35], we also examined the effect of AZD0530 treatment on neuroinflammation in PS19 mice using anti-Iba1 and GFAP antibodies, general makers for microgliosis and astrocytosis, respectively. At 9 months of age, despite an evident increase in Tau pathology, we did not observe a significant increase in Iba1 or GFAP immunoreactivities in the hippocampus of PS19 mice under a low magnification condition (Supplementary Fig. S3). We thus analyzed specific subregions of the hippocampus (i.e. the CA1, CA3, and DG) using high-resolution spinning disc confocal microscopy. While there was no difference in Iba1-immunoreactivity between WT and PS19 mice in these subregions, co-immunostaining using an antibody against CD68, a maker for activated microglia, revealed a significant increase in CD68-immunoreactivity within the Iba1-immunoreactive area of the hippocampal CA3 area in PS19 mice. Consistent with the results showing a reduction in Tau pathology, AZD0530 treatment almost completely prevented the increase in CD68 immunoreactivity in PS19 mice (Fig. 3a, b). Using high-resolution imaging, we also found a moderate but significant increase in GFAP-immunoreactive area in the DG, but not the other subregions, of hippocampus of Vehicle-treated PS19 mice at 9 months of age. Importantly, AZD0530 treatment also significantly attenuated the astrocytosis in PS19 mice (Fig. 3c, d). These results indicate that AZD0530 treatment prevents neuroinflammation in the hippocampus of PS19 mice.

### Synapse loss in PS19 Transgenics is prevented by AZD0530

To further investigate the mechanisms by which AZD0530 treatment rescues behavioral deficits in PS19 mice, we examined the effects of AZD0530 treatment on presynaptic degeneration observed in PS19 mice. Similar to previous studies using immunostaining for synaptophysin [1, 35, 71], there was a significant reduction in immunoreactivity of SV2A, a presynaptic protein, in the CA3 area of hippocampus of PS19 mice compared to that of WT mice at 9 months of age (Fig. 3e, f). AZD0530 treatment fully rescued the reduction in SV2A immunoreactivity in the CA3 area of hippocampus of PS19 mice (Fig. 3e, f). Thus, chronic Fyn kinase inhibition prevents phospho-Tau accumulation, gliosis and synapse loss, thereby permitting rescue of memory function in this model.

### Fyn inhibition does not Alter Tyr18 phosphorylation of tau in PS19 mice

**Fig. 4:**
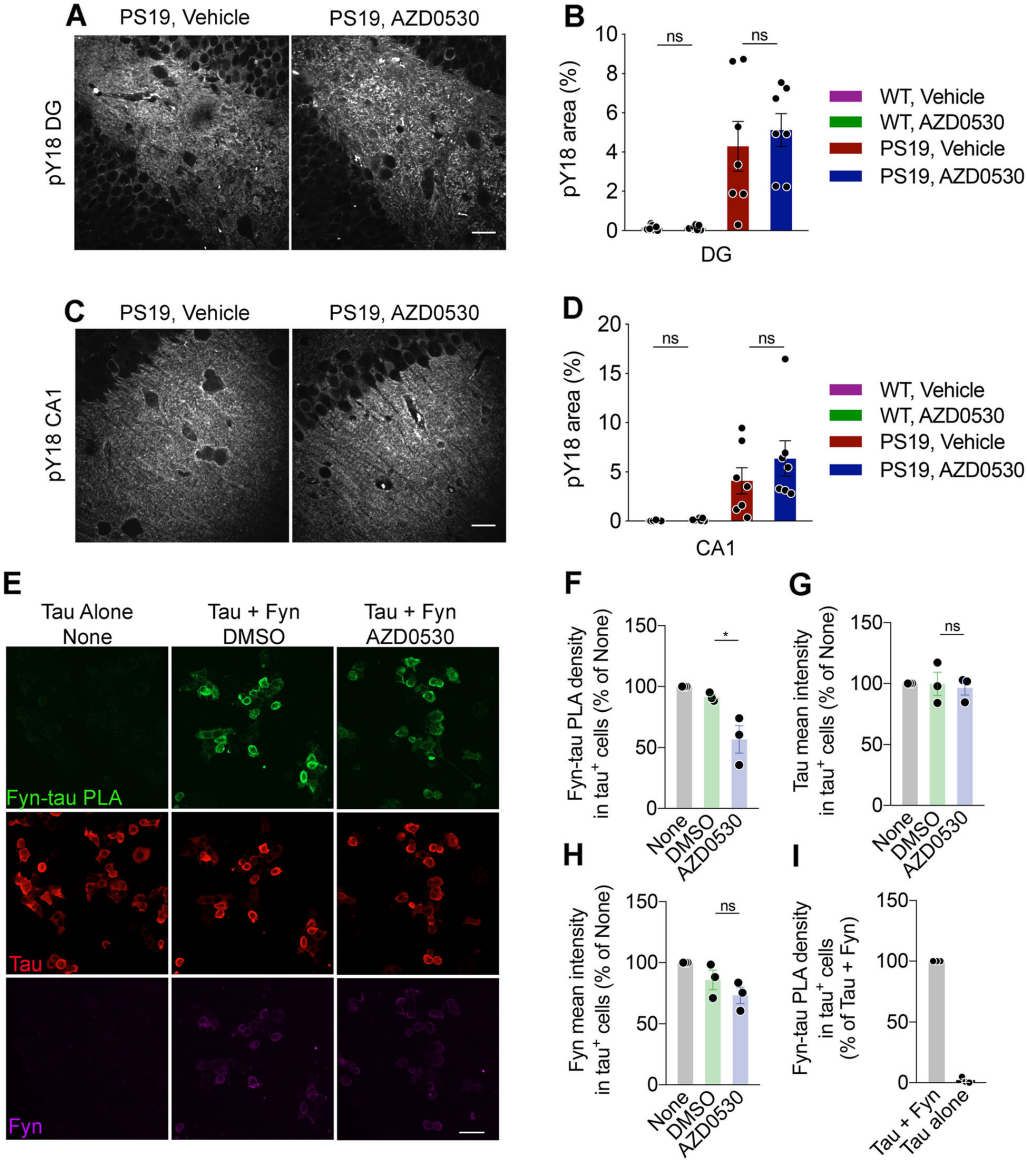
Fyn Kinase Inhibitor Does Not Alter Tau pY18 but Does Reduce Fyn/Tau Colocalization. **(a)** Representative images of immunofluorescent staining for pY18 in the dentate gyrus (DG) of the hippocampus in 9-month-old PS19 mice after 7 months of AZD0530 or Vehicle treatment. Scale bar, 20 μm. **(b)** Quantification of pY18-positive area (%) in the dentate gyrus (DG) of the hippocampus in 9-month-old PS19 and WT mice after 7 months of treatment. Data are mean ± SEM. *n* = 7–10, each dot is the average of three sections from one mouse. One-way ANOVA with Sidak’s multiple comparisons test. **(c)** Representative images of immunofluorescent staining for pY18 in the CA1 of the hippocampus in 9-month-old PS19 mice after 7 months of treatment. Scale bar, 20 μm. **(d)** Quantification of pY18-positive (%) area in the CA1 of the hippocampus in 9-month-old PS19 and WT mice after 7 months of treatment. Data are mean ± SEM. *n* = 7–10, each dot is the average of three sections from one mouse. Each point is the average of 3 slices from the same animal. One-way ANOVA with Sidak’s multiple comparisons test. **(e)** Representative immunofluorescent images of PLA from HEK-293 T cells expressing human Fyn and Tau treated with 2 μM AZD0530 in DMSO or DMSO (control) for 24 h. Fyn-Tau PLA in green are sites of Fyn and Tau interaction. Tau-positive area are in red and Fyn-positive area are in magenta. Scale bar, 20 μm. **(f)** Quantification of the area of Fyn- Tau PLA density (%) within Tau-positive area of HEK-293 T cells expressing human Fyn and Tau, normalized to the condition with no treatment. Data are mean ± SEM. *n* = 3. Each point represents the average of four images taken per experimental condition. *p < 0.05; Unpaired t-test. **(g)** Quantification of the mean intensity of Tau (%) within Tau-positive area of HEK-293 T cells, normalized to the condition with no treatment. Data are mean ± SEM. *n* = 3. Each point represents the average of four images taken per experimental condition. Unpaired t-test. **(h)** Quantification of mean intensity of Fyn (%) within Tau-positive area of HEK-293 T cells, normalized to the condition with no treatment. Data are mean ± SEM. *n* = 3. Each point represents the average of four images taken per experimental condition. Unpaired t-test. **(i)** Quantification of the percent Fyn-Tau PLA density of HEK-293 T cells expressing only human tan in Tau-positive cells, normalized to the percent density in Tau- and Fyn- transfected HEK-293 T cells. Data are mean ± SEM. *n* = 3. Each point represents the average of four images taken per experimental condition

Tau is both a binding partner and substrate of Fyn kinase. Fyn phosphorylates Tau at Tyr18 [4, 31, 33]. To better understand the mechanism by which Fyn inhibition attenuates Tau pathology, we examined the effects of AZD0530 treatment on pY18 levels in PS19 mice after AZ0530 treatment for 7 months. There was a clear increase of pY18 immunoreactivity for PS19 versus WT mice. Unexpectedly, staining with the anti-pY18 antibody showed no significant difference in the DG or CA1 of the hippocampus between AZD0530-treated and vehicle-treated PS19 mice (Fig. 4a-d). Thus, chronic Fyn inhibition does not affect phosphorylation levels at Tyr18 even though it strongly suppresses AT8 (phospho-Ser202/Thr205) and PHF1 (phospho-Ser396/Ser404) pathologies in the PS19 transgenic mice.

### Fyn inhibition decreases Fyn and tau interaction

Given the absence of AZD0530 effect on pY18 levels, we considered whether AZD0530 treatment might alter the physical interaction of Fyn with Tau secondary to altered Fyn activation state. We examined a proximity ligation assay (PLA) in HEK-293 T cells expressing human Fyn and Tau [51]. Cells treated with AZD0530 for 24 h exhibited significantly less Fyn-Tau PLA density than cells that were treated with vehicle control (Fig. 4e, f). AZD0530 treatment had no significant effect on total levels of Tau (Fig. 4g) and Fyn (Fig. 4h) in the same area; only their proximity was altered by drug treatment. Confirming assay specificity, the Fyn-Tau PLA signal was not detectable when only human Tau expression vector was transfected in the HEK-293 T cells (Fig. 4i). These results suggest that AZD0530 treatment acts to decrease the interaction between Fyn and Tau, with subsequent reduction in Ser/Thr phosphorylation of Tau.

### Fyn inhibition improves memory function in rmTBI/stress model

**Fig. 5:**
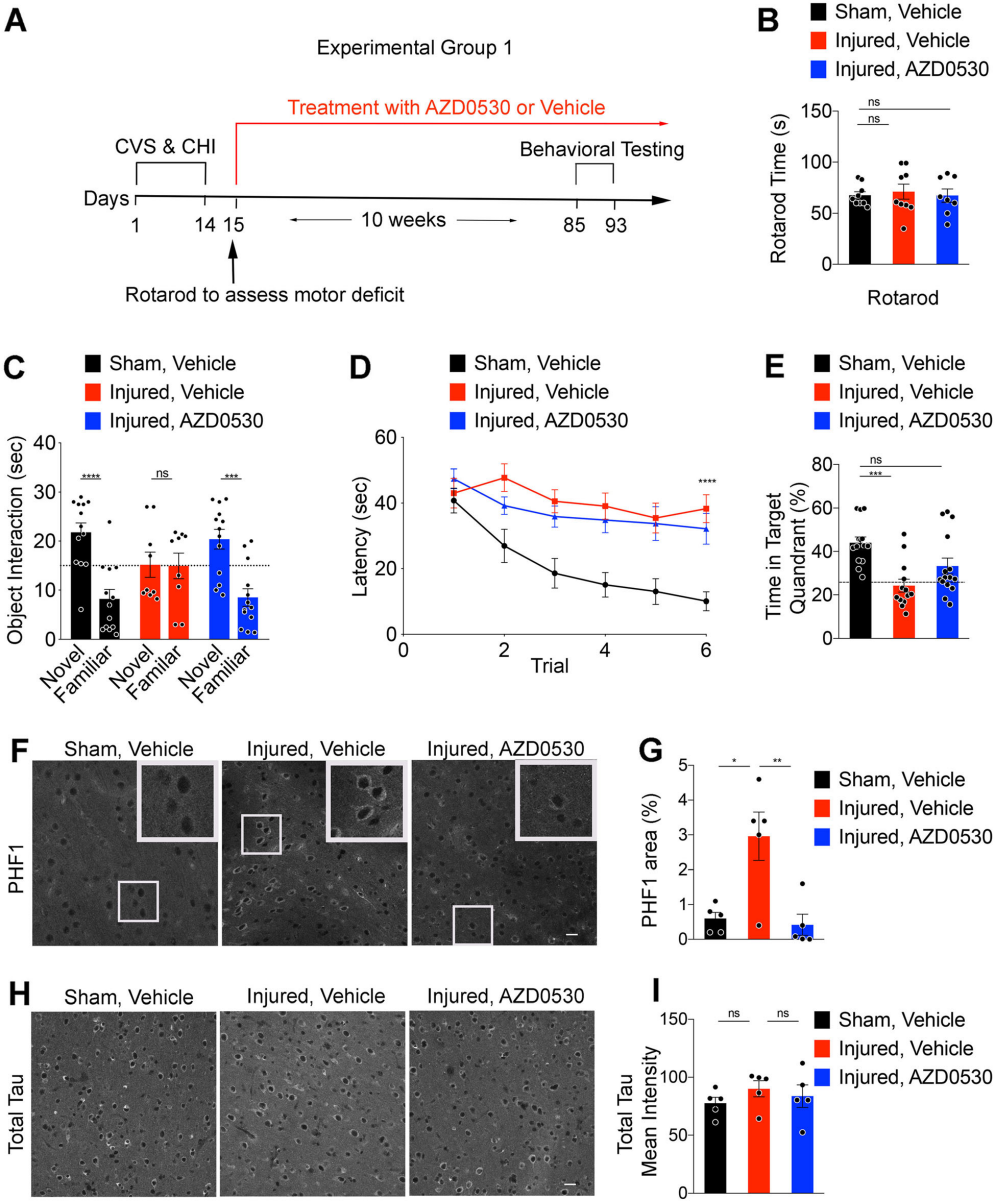
Fyn Inhibition Rescues Memory Deficits and Prevents Phospho-Tau Accumulation after Repeated Mild Head Injury Combined with Chronic Stress. **(a)** Timeline for mice undergoing 14 days of chronic variable stress (CVS) and closed head injury (CHI) or Sham CVS & CHI paradigm. On Day 15, Rotarod testing was done to assess motor impairment in a subset of mice. The mice were treated with either AZD0530 (5 mg/kg/d) or Vehicle treatment for 10 weeks starting 24 h after the final day of injury. This was followed by one week of behavioral testing at 7 months of age, including novel object recognition test and Morris water maze prior to perfusion and immunohistochemistry. **(b)** Rotarod prior to the treatment using the WT Sham and Injured groups at 4.5 months of age. One-way ANOVA, *p* > 0.05. Data are mean ± SEM. *n* = 8–9 /group, each dot is one mouse. **(c)** Novel object recognition test of 7-month-old WT mice from Sham Vehicle-treated (SV), Injured Vehicle-treated (IV), and Injured AZD0530-treated (IA) groups. Data are mean ± SEM. *n* = 9–13/group, each dot represents one mouse. Two-way ANOVA, *p* = 0.007 for interaction of group with object; Sidak’s multiple comparison test: Novel vs Familiar for Sham Vehicle (SV), ****p < 0.0001; Injured Vehicle (IV), *p* = 0.99; Injured AZD (IA), ***p = 0.0002. **(d)** Latency to reach a hidden platform in Morris water maze across 6 blocks of 4 swims of 7-month-old WT mice from SV, IV, and IA groups. Data are mean ± SEM. *n* = 14–17 /group. Repeated measures one-way ANOVA with Tukey’s multiple comparisons test: SV vs IV, ****p < 0.0001; IV vs IA, *p* = 0.98; SV vs IA, ****p < 0.0001. **(e)** Morris water maze probe trial showing time in the Target quadrant of 7-month-old WT mice from SV, IV, and IA groups. Dashed line indicates random chance performance of 25% in the target quadrant. Data are mean ± SEM. *n* = 13–15 /group, each dot is one mouse. Two-tailed Wilcoxon signed rank test for non-Gaussian distribution versus random chance: SV, ***p = 0.0001; IV, *p* = 0.47; IA, *p = 0.046. One way ANOVA with Tukey’s multiple comparisons test: SV vs IV, *p* = 0.0003; IV vs IA, *p* = 0.12; SV vs IA, *p* = 0.05. **(f)** Representative images of immunofluorescent staining for PHF1 of coronal cerebral cortex sections within 0.5–1 mm medial to the site of injury in 7.5-month-old WT mice from SV, IV, and IA groups. Boxed area is shown at higher magnification inset. Scale bar, 20 μm. **(g)** Quantification of PHF1-positive area within 0.5–1 mm medial to the site of injury in 7.5-month-old WT mice from SV, IV, and IA groups. Data are mean ± SEM. *n* = 5 /group, each dot is one mouse. One way ANOVA with Tukey’s multiple comparisons test: SV vs IV, *p = 0.0077; IV vs IA, **p = 0.0046. **(h)** Representative images of immunofluorescent staining for total Tau in the cortical sections at the same region as F in 7.5-month-old WT mice from SV, IV, and IA groups. Scale bar, 20 μm. **(i)** Quantification of total Tau mean intensity at the same region as F in 7.5-month-old WT mice from SV, IV, and IA groups. Data are mean ± SEM. *n* = 5 /group, each dot is one mouse. One way ANOVA with Tukey’s multiple comparisons test

Having observed a benefit of Fyn inhibition in the PS19 transgenic model, we sought to extend our analysis to a traumatic Tauopathy. In order to mimic conditions resembling those related to combat and those associated with CTE, we exposed mice to daily mild closed head injury and chronic variable stress for 14 consecutive days. The parasagittal injury site alternated right to left on different days. Preliminary work demonstrated that the exposure to injury plus stress is synergistic in establishing persistent neurological deficits (A.F.Z, unpublished). At the end of the 14 day induction period, motor deficits were minimal, as revealed by Rotarod performance indistinguishable from the Sham group, which received similar extent of handling and anesthesia (Fig. 5a, b). Furthermore, there was minimal evidence of tissue damage or neuronal loss in this model as evidenced by cresyl violet stain or anti-NeuN immunohistology (Supplementary Fig. S4A-C).

One experimental group received 5 mg/kg/day of AZD0530 or Vehicle beginning 24 h after the final day of injury on Day 15 for a period of 10 weeks (Fig. 5a-e). The daily dose was divided into twice a day oral gavage administration as in previous AD-related studies [27]. While still receiving AZD0530 treatment or Vehicle, learning and memory were assessed. The Injured Vehicle group demonstrated profound deficits in novel object recognition test with no ability to distinguish novel versus familiar objects as opposed to Sham mice with a robust preference for novel objects (Fig. 5c). The Injured AZD0530 group recognized familiar objects as successfully as Sham Vehicle mice (Fig. 5c). Spatial learning and memory were assessed as described for PS19 mice. Despite the lack of a motor deficit, the Injured Vehicle group failed to learn the hidden platform location over 6 blocks of 4 swim trials, and was significantly impaired relative to Sham Vehicle mice (Fig. 5d). The Injured mice treated with AZD0530 continued to show severely impaired learning relative to Sham Vehicle (Fig. 5d). In the probe trial 1 day after the learning trials, the Injured Vehicle mice performed indistinguishably from random chance and significantly worse than Sham, consistent with an absence of memory for the previous platform location (Fig. 5e). In contrast, the Injured AZD0530 group recalled the hidden platform location significantly greater than chance, and their performance was not significantly different from the Sham group (Fig. 5e). Thus, AZD0530 treatment beginning 24 h after the 2-week injury epoch, fully rescued novel object recognition memory and partially rescued spatial memory performance.

We considered whether the benefit of Fyn kinase might extend to chronic injury conditions. A second group of mice received AZD0530 treatment beginning on Day 121, 107 days after the final day of injury and treatment continued for 10 weeks (Supplementary Fig. S5A). Spatial learning and memory deficits in the Injured Vehicle group remained pronounced more than 4 months post injury reflecting the chronic nature of this injury model (Supplementary Fig. S5B, S5C). As compared to the first cohort, for which treatment began 1 day after the 2-week injury period, there was no evidence of improved memory in the probe trial of the Morris water maze after the learning trials (Supplementary Fig. S5C). We conclude that the benefit of Fyn kinase inhibition in this model of combined rmTBI/Stress is limited to the subacute post-injury phase.

### AZD0530 treatment reduces Tauopathy after rmTBI/stress

In the PS19 transgenic model of Tauopathy, the benefit of AZD0530 treatment correlated with reduced phospho-Tau accumulation histologically. We examined tissue from the subacute rmTBI/Stress mice by PHF1 immunostaining in the cerebral cortex 0.5–1.0 mm medial to the injury site. PHF1 immunoreactivity area was significantly increased in the Injured Vehicle group compared to the Sham Vehicle group. Similar to the results from the PS19 transgenic experiment, the Injured AZD0530 samples exhibited dramatically reduced PHF1 immunoreactive area (Fig. 5f, g). The PHF1-positive Tau was not stained with thioflavin S (data not shown), demonstrating that the rmTBI/Stress-induced Tau pathology had not fully developed into tangle-like fibrillary inclusions. In contrast to phospho-Tau, total Tau immunoreactivity area was not altered by injury or Fyn kinase inhibitor treatment (Fig. 5h, i). We also examined microgliosis and astrogliosis by anti-Iba1 and GFAP staining, respectively, in the perilesional area (Supplementary Fig. S6). There was no significant difference between Injured and Sham groups, reflecting the minor and chronic nature of the injury.

### Fyn inhibition by AZD0530 treatment prevents tau seeding

**Fig. 6:**
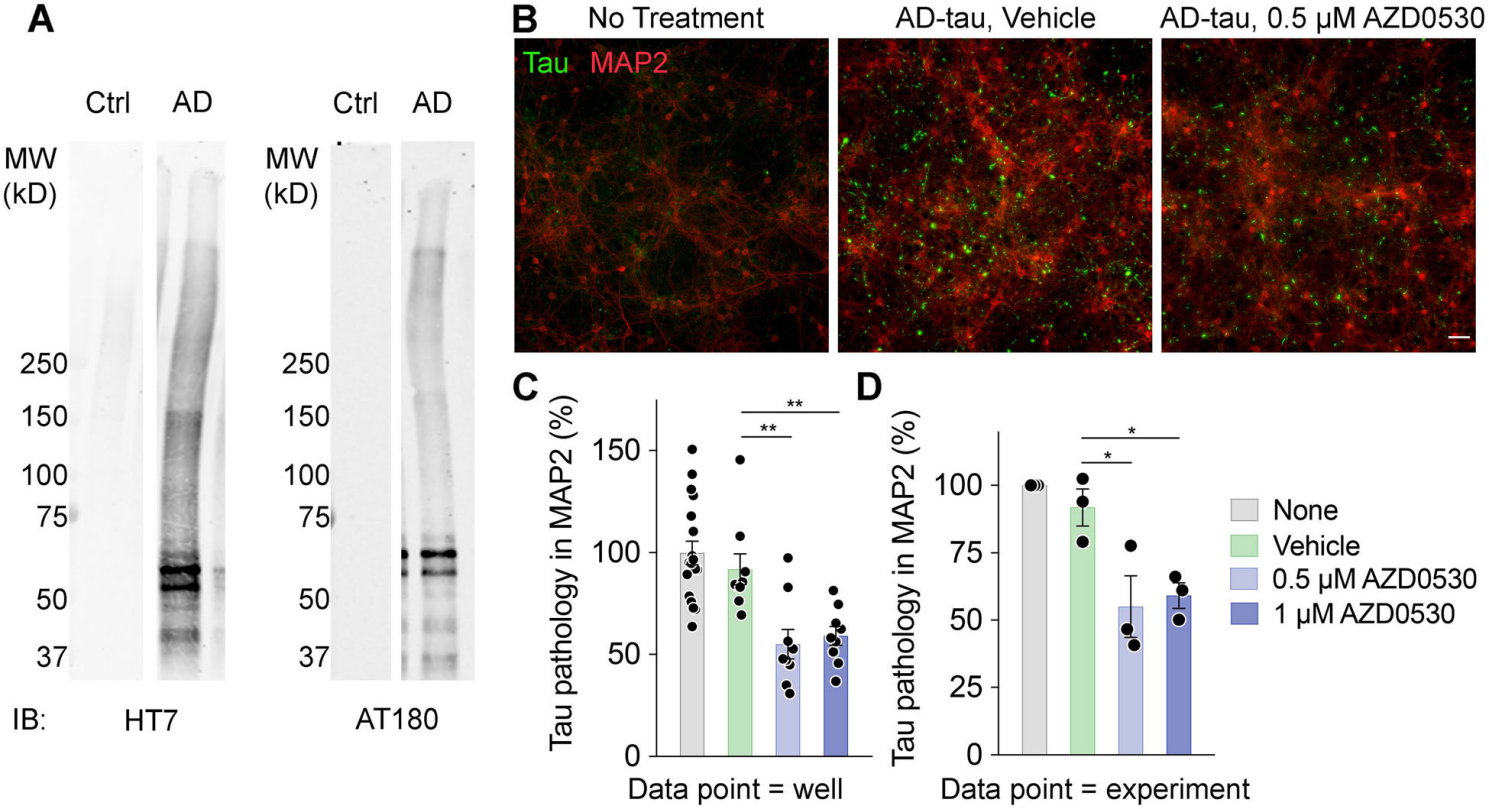
Fyn Inhibition Prevents Tau Seeding in Neurons. **(a)** Representative blots of Tau extracts using HT7 and AT180 antibodies to show prominent Tau bands between 50 and 75kD from human AD patient as compared to a healthy control. **(b)** Immunostaining of endogenous mouse Tau and MAP 2 in WT neurons fixed with methanol at DIV21 after AD-Tau seeding and treatment at DIV7. Left panel shows neurons with no AD-Tau seeding nor treatment. Middle panel shows neurons with AD-Tau seeding and treatment with vehicle (water). Right panel shows neurons with AD-Tau seeding and treatment with 0.5 μM AZD0530. **(c)** Quantification of percentage of mouse tau-positive area within MAP 2-positive area. Neurons were either not seeded with Tau; only seeded with AD-Tau; seeded with AD-Tau and treated with water as vehicle; seeded with AD-Tau and 0.5 μM of AZD0530; or seeded with AD-Tau and 1 μM of AZD0530. The background signal from images of neurons without AD-Tau treatment was subtracted. Experiments were performed in triplicate. Each data point represents the average of values obtained from four images taken from one well. Data are mean ± SEM. *n* = 9–18 per experimental condition. **p < 0.01; One-way ANOVA with Dunnett’s multiple comparisons test. **(d)** Same quantification as C, except each data point represents the average of three wells of each condition from one experiment. Data are mean ± SEM. *n* = 3. *p < 0.05; Repeated measures one-way ANOVA with Dunnett’s multiple comparisons test

Inhibition of Fyn by AZD0530 may reduce Tau pathology in a cell autonomous manner and/or by limiting of Tau propagation between cells [15, 19] and between regions in the brain [10]. The phenomenon of tau spreading has been suggested to play a key role in the progression of tauopathies [67]. To investigate whether AZD0530 treatment inhibits Tau spreading, we performed a Tau seeding assay using mouse primary cultured neurons as reported [19]. Tau was extracted from an AD patient (Fig. 6a) and the AD-Tau was seeded onto WT neurons at DIV7. Consistent with the previous study [19], at DIV21, neurons that were seeded with AD-Tau and treated with vehicle or without treatment displayed higher levels of aggregation of endogenous mouse Tau (detected by T49 mouse Tau-specific antibody in methanol-fixed neurons) (Fig. 6b-d). Importantly, neurons that were seeded with AD-Tau and treated with AZD0530 at 0.5 and 1 μM concentrations had significantly lower levels of induced aggregation. These results suggest that Fyn inhibition by AZD0530 treatment prevents Tau spreading between cells.

## Discussion

The primary finding of the current study is the ability of Fyn kinase inhibition to prevent Tau accumulation and memory deficits in both transgenic and traumatic models of Tauopathy in mice. In the P301S model, chronic treatment initiated in early adulthood reduced subsequent gliosis and synapse loss as well as accumulation of phospho-Tau. This attenuation of pathology resulted in preservation of learning and memory performance over 6 months. Our traumatic injury model combined repeated CHI with CVS to create persistent learning and memory deficits with no detectable motor impairment. In this rmTBI/Stress model, post-injury Fyn inhibition reduced focal phospho-Tau accumulation, fully rescuing object recognition and improving spatial memory function.

Blocking Fyn kinase activation with AZD0530 reduces autophosphorylation of the enzyme in the activation loop, and the conformational changes associated with enzyme activation are prevented [8]. In this way, the inhibition of kinase activity with the ATP-competitive inhibitor AZD0530 blocks both phosphorylation and protein interactions dependent on the activated enzyme conformation. Tau is both a binding partner and a substrate of Fyn kinase. Occupancy with AZD0530 has the potential to reduce both phosphorylation of Fyn substrates as well as complex formation with Fyn partners. Tau accumulation is accompanied by phosphorylation of Ser/Thr residues introduced by other kinases, and common pathology-associated epitopes include Ser202/Thr205 detected by AT8 antibody and Ser396/Ser404 detected by PHF1 antibody. The reduction of these epitopes is an indirect result of Fyn kinase inhibition. The change in Ser/Thr phosphorylation and accumulation may be secondary to transiently altered Tyr18 phosphorylation, or more likely to altered Fyn/Tau binding with shifted subcellular localization and changed access to Ser/Thr kinases [24]. Our studies revealed no change in phospho-Y18 Tau levels in AZD0530-treated PS19 samples despite reduction in AT8 and PHF1 accumulation. Furthermore, in WT mice with or without trauma, this phospho-Y18 epitope was not detectable. Rather, the binding of AZD0530 to Fyn blocks the interaction between Fyn and Tau, as observed in the decreased Fyn-Tau localization in the PLA. This, in turn, may suspend Fyn-mediated mis-localization of Tau to the post-synaptic area and prevent further Tau spreading between neurons. It is clear that the net result of Fyn kinase inhibition is reduced Ser/Thr phosphorylation and accumulation of Tau.

AZD0530 inhibits all Src family kinases with similar potency, but has minimal affinity for other kinase families. With regard to CNS expression, Fyn and Src, and to a lesser extent Yes, are the most prominent family members. Of these three, previous studies of Amyloid-ß, Tau and AD signaling have shown a specific role for Fyn [6, 7, 24, 29, 30, 48, 63, 64]. Therefore, while AZD0530 is selective for Src family kinases, its activity with respect to Tauopathy is likely mediated primarily via Fyn.

In the present study, we have shown that Fyn inhibition alters Tau phosphorylation and accumulation, which is associated with improved behavior. However, Fyn has multiple interactors and substrates in addition to Tau. These other substrates, including NMDA-Rs, may contribute to the rescue observed by AZD0530 [59]. For the PS19 model, aberrant Tau is the driving factor in triggering the impaired behavior, so this favors a direct role for Tau interaction with Fyn in the benefit of AZD0530.

The clinical history for cases of CTE, and PTSD in combat veterans, typically includes both mild repetitive head injury and chronic unpredictable stress. A progressive Tauopathy with devastating behavioral and cognitive deficits has been described [14, 37]. We sought to model this condition by combining daily mild CHI with CVS in mice over 2 weeks. It is clear that this paradigm produces profound learning and memory deficits and includes accumulation of phospho-Tau epitopes. Much like the clinical conditions, there is minimal if any motor dysfunction. However, we have not observed the late development of progressive and widespread degeneration in such mice that fully recapitulates CTE. This may relate to differences in the lifespan of the mouse and/or to the organization and expression of the *MAPT* locus across species. In this rmTBI/Stress model, Fyn kinase inhibition rescued object recognition memory deficits and reduced spatial memory deficits. While behavioral deficits in this model are not likely to depend exclusively on Tauopathy, the model was chosen because it includes two clinically relevant factors (i.e. CHI and CVS) and produces a prolonged behavioral deficit as well as the changes in phospho-Tau described above. Our goal in the rmTBI/Stress studies was not to analyze the basic pathophysiology of the model but rather to assess the responsiveness to Fyn inhibition. Although Tau pathology is observed in this model, this study does not confirm that Tau pathology is the cause of the behavioral deficit. Given AZD0530 responsiveness and the effect of AZD0530 in PS19 mice and tau seeding in neuronal culture, we hypothesize that behavioral benefit is due to reduced PHF1-positive Tau but this must be confirmed in future studies with Tau-deficient mouse.

A key aspect of any potential therapeutic intervention is its timing relative to disease diagnosis, symptoms and progression. In the PS19 transgenic model, the Fyn kinase benefit was observed in a prophylactic mode initiated prior to the onset of Tau accumulation. For the rmTBI/Stress model, we initiated treatment a full 24 h after the 2 week injury/stress paradigm was complete in a therapeutic mode, and observed robust benefit. However, the time window for effective Fyn kinase intervention does not appear open-ended, since treatment initiated 3–4 months after the injury period did not reverse well established deficits.

It is clear from these studies that modulating the activation state of a Tau partner, Fyn kinase, alters the course of both genetic and traumatic Tauopathy. Specifically, reducing Fyn activation leads to less phospho-Tau accumulation, with a normalization of glial activity, synapse density and memory function. Moreover, effective intervention can be achieved even when delayed by a full 24 h after an extended 2 week injury/stress exposure.

## Conclusion

Fyn inhibition with AZD0530 prevents the development of Tau pathology in both PS19 transgenic and rmTBI/Stress mouse models. The kinase inhibitor blocks the activation of Fyn and prevents its interactions with Tau. As a result of AZD0530 treatment in the PS19 mouse model, there is a decrease in phospho-Tau accumulation, prevention of gliosis, rescue of synapse density, and prevention of memory loss. AZD0530 treatment in a rmTBI/stress mouse model with CVS and repeated mild CHI reduced phospho-Tau accumulation and prevented memory deficits. These changes are observed as a result of a decrease in Fyn-Tau localization rather than a decrease in the phosphorylation of Tyr18. Because AZD0530 has been tolerated chronically in clinical trials, there is an opportunity to examine the benefit of Fyn kinase inhibition in Tauopathy conditions.

## Supplementary information

**Additional file 1: Figure S1.** Reduced Fyn Activation in Mice Fed AZD0530-Containing Food. (A) Representative blots using anti-pY416, Fyn, and ß-actin antibodies of the RIPA-soluble fraction of the hippocampus of WT mice treated with Vehicle or AZD0530 for 9 months. (B) Quantification of pY416-immunoreactive bands from the immunoblot from A by densitometric analysis. The bands indicated by an arrow in A were quantified. The band intensity was normalized to that of ß-actin and then normalized to the mean of the Vehicle-treated WT group. Data are represented as mean ± SEM. *n* = 6 /group. *p < 0.05; t-test. (C) Quantification of Fyn-immunoreactive bands from the immunoblot in A by densitometric analysis. The band intensity was normalized to that of ß-actin and then normalized to the mean of the Vehicletreated WT group. Data are represented as mean ± SEM. *n* = 6 /group. t-test. **Figure S2.** Behavioral Tests of PS19 or WT Mice Treated with AZD0530 or Vehicle (A) Morris water maze distance traveled for forward and reverse swims in 8-month-old PS19 and WT mice after 6 months of treatment. Pathlength is measured as the total distance traveled (in cm) before the mouse reaches the submerged platform. Data are mean ± SEM. *n* = 11-15 /group. One-way ANOVA. (B) Morris water maze latency to target for forward and reverse swims in 8-month-old PS19 and WT mice after 6 months of treatment. The latency was measured as the time for the mouse to find a submerged platform in a forward and a reverse swim after a platform relocation. Data are mean ± SEM. *n* = 11-15 /group. One-way ANOVA. (C) Morris water maze visible platform trial after reverse swim. Latency is measured as the average amount of time the mouse takes to reach the flagged platform in an average of 12 trials or until the latency has plateaued for 3 trials, whichever comes first. Data are mean ± SEM. *n* = 11-15 /group, each dot from one mouse. *p < 0.05, One-way ANOVA with Holm-Sidak’s multiple comparisons test. (D) Rotarod trials in 8-month-old PS19 and WT mice of the prophylactic cohort. Latency to fall is measured as the time it takes to fall from the rotating, accelerating rod. Each data represents the average of 5 trials for one mouse. Data are mean ± SEM. *n* = 18-19 /group. One-way ANOVA. **Figure S3.** Low Magnification Survey of Gliosis in PS19 Mice Unaffected by AZD0530. (A) Representative images of Iba1 immunostaining in the hippocampus in 9-month-old PS19 and WT mice after 7 months of treatment. Scale bar, 100 μm. (B) Quantification of Iba1-positive area (%) in the hippocampus in 9-month-old PS19 and WT mice collected after 7 months of treatment. Data are mean ± SEM. *n* = 7-10 /group. One-way ANOVA. (C) Representative images of immunofluorescent staining GFAP in the hippocampus in 9-month-old PS19 and WT mice after 7 months of treatment. Scale bar, 100 μm. (D) Quantification of GFAP-positive area (%) in the hippocampus in 9-month-old PS19 and WT mice collected after 7 months of treatment. Data are mean ± SEM. *n* = 7-10 /group. One-way ANOVA. **Figure S4.** Minimal Tissue Damage or Neuronal Loss after rmTBI/Stress. (A) Representative cresyl violet stained images of Sham Vehicle-treated (Sham) and Injured Vehicletreated (Injured) of the cortex and hippocampal regions containing the injury site collected more than 3 months after injury. (B) Representative NeuN stained images of Sham Vehicle-treated (Sham) and Injured Vehicletreated (Injured) coronal sections of cerebral cortex within 0.5-1 mm medial to the site of injury, using 20X magnification. Scale bar, 20 μm. (C) Representative NeuN stained images of sham vehicle treated (Sham) group and injured vehicle treated (Injured) coronal sections from the CA1 region of the hippocampus within 1 mm of the site of injury, using 20X magnification. Scale bar, 20 μm. **Figure S5.** Fyn Inhibitor Treatment of Chronic rmTBI/Stress Mice. (A) Timeline for a second cohort of mice that underwent a similar 14 days of chronic variable stress (CVS) plus closed head injury (CHI) or Sham CVS & CHI paradigm, and then starting on Day 121 were treated with either AZD0530 (5 mg/kg/d) or Vehicle for 10 weeks. The mice subsequently underwent Morris water maze testing at 11 months of age. (B) Latency to reach a hidden platform in reverse Morris water maze for 11-month-old WT mice from Sham Vehicle-treated (SV), Injured Vehicle-treated (IV), and Injured AZD0530-treated (IA) groups. Latency is measured as the time it takes for the mouse to reach the hidden platform. Both Injured groups exhibited longer latency to the hidden platform compared to the Sham group, but the two Injured groups were not significantly different from one another. Data are mean ± SEM. *n* = 8-26 /group. Twoway ANOVA, ****p < 0.0001; Tukey’s multiple comparisons test. (C) Morris water maze probe trial performed 24 hours after training trials in B for 11-month-old WT mice from SV, IV, and IA groups. Neither mice from IV nor IA groups demonstrated preference towards the target quadrant. Dashed line indicates random chance performance of 25% in the target quadrant. Data are mean ± SEM. *n* = 8-26 /group, each dot is one mouse. Two-tailed Wilcoxon signed rank test for non-Gaussian distribution versus random chance: SV, ***p = 0.0001; IV and IA, n.s., *p* > 0.05 . One way ANOVA, Tukey’s multiple comparisons test: SV vs IV, ****p < 0.0001; IV vs IA, n.s., *p* > 0.05; SV vs IA, ****p < 0.0001. **Figure S6.** Minimal Microgliosis and Astrogliosis Months after rmTBI/Stress Unaffected by Fyn Inhibitor. (A) Representative images of immunofluorescent staining Iba1 of cortical sections in the same region as Fig. 5f,h in 7.5-month-old WT mice from Sham Vehicle-treated (SV), Injured Vehicle-treated (IV), and Injured AZD0530-treated (IA) groups. Scale bar, 20 μm. (B) Quantification of Iba1-positive area (%) in 7.5-month-old WT mice from SV, IV, and IA groups. Data are mean ± SEM. *n* = 5 / group, each dot is one mouse. One way ANOVA with Dunnett’s multiple comparisons test, (C) Representative images of immunofluorescent staining GFAP of cortical sections in the same region as in Fig. 5f, h in 7.5-month-old WT mice from SV, IV, and IA groups. Scale bar, 20 μm. (D) Quantification of GFAP-positive area (%) in 7.5-month-old WT mice from SV, IV, and IA groups. Data are mean ± SEM. *n* = 5 / group, each dot is one mouse. One way ANOVA with Tukey’s multiple comparison test. **Table S1.** Mouse Cohorts

## Data Availability

ImageJ macros, CellProfiler pipelines and original data generated from this study are available upon request.

## Abbreviations

AD: Alzheimer’s disease
CTE: Chronic Traumatic Encephalopathy
CHI: Close Head Injury
CVS: Chronic Variable Stress
DG: Dentate Gyrus
DIV: Days In Vitro
PLA: Proximity Ligation Assay
PTSD: Post-Traumatic Stress Disorder
TBI: Traumatic Brain Injury
rmTBI: Repetitive mild Traumatic Brain Injury
WT: Wild-type
