# Supplementary material for: Fyn kinase inhibition reduces protein aggregation, increases synapse density and improves memory in transgenic and traumatic Tauopathy
Source: Acta Neuropathol Commun. 2020 Jul 1;8:96. doi: 10.1186/s40478-020-00976-9 (PMC7329553; doi:10.1186/s40478-020-00976-9)
Supplement: Supplementary file 1 — Additional file 1: Figure S1. Reduced Fyn Activation in Mice Fed AZD0530-Containing Food. (A) Representative blots using anti-pY416, Fyn, and ß-actin antibodies of the RIPA-soluble fraction of the hippocampus of WT mice treated with Vehicle or AZD0530 for 9 months. (B) Quantification of pY416-immunoreactive bands from the immunoblot from A by densitometric analysis. The bands indicated by an arrow in A were quantified. The band intensity was normalized to that of ß-actin and then normalized to the mean of the Vehicle-treated WT group. Data are represented as mean ± SEM. n = 6 /group. *p < 0.05; t-test. (C) Quantification of Fyn-immunoreactive bands from the immunoblot in A by densitometric analysis. The band intensity was normalized to that of ß-actin and then normalized to the mean of the Vehicletreated WT group. Data are represented as mean ± SEM. n = 6 /group. t-test. Figure S2. Behavioral Tests of PS19 or WT Mice Treated with AZD0530 or Vehicle (A) Morris water maze distance traveled for forward and reverse swims in 8-month-old PS19 and WT mice after 6 months of treatment. Pathlength is measured as the total distance traveled (in cm) before the mouse reaches the submerged platform. Data are mean ± SEM. n = 11-15 /group. One-way ANOVA. (B) Morris water maze latency to target for forward and reverse swims in 8-month-old PS19 and WT mice after 6 months of treatment. The latency was measured as the time for the mouse to find a submerged platform in a forward and a reverse swim after a platform relocation. Data are mean ± SEM. n = 11-15 /group. One-way ANOVA. (C) Morris water maze visible platform trial after reverse swim. Latency is measured as the average amount of time the mouse takes to reach the flagged platform in an average of 12 trials or until the latency has plateaued for 3 trials, whichever comes first. Data are mean ± SEM. n = 11-15 /group, each dot from one mouse. *p < 0.05, One-way ANOVA with Holm-Sidak’s multiple comparisons test. (D) Rotarod trials in [file 40478_2020_976_MOESM1_ESM.pdf]

SUPPLEMENTARY INFORMATION

Supplementary Figures S1-S6 and Supplementary Table S1

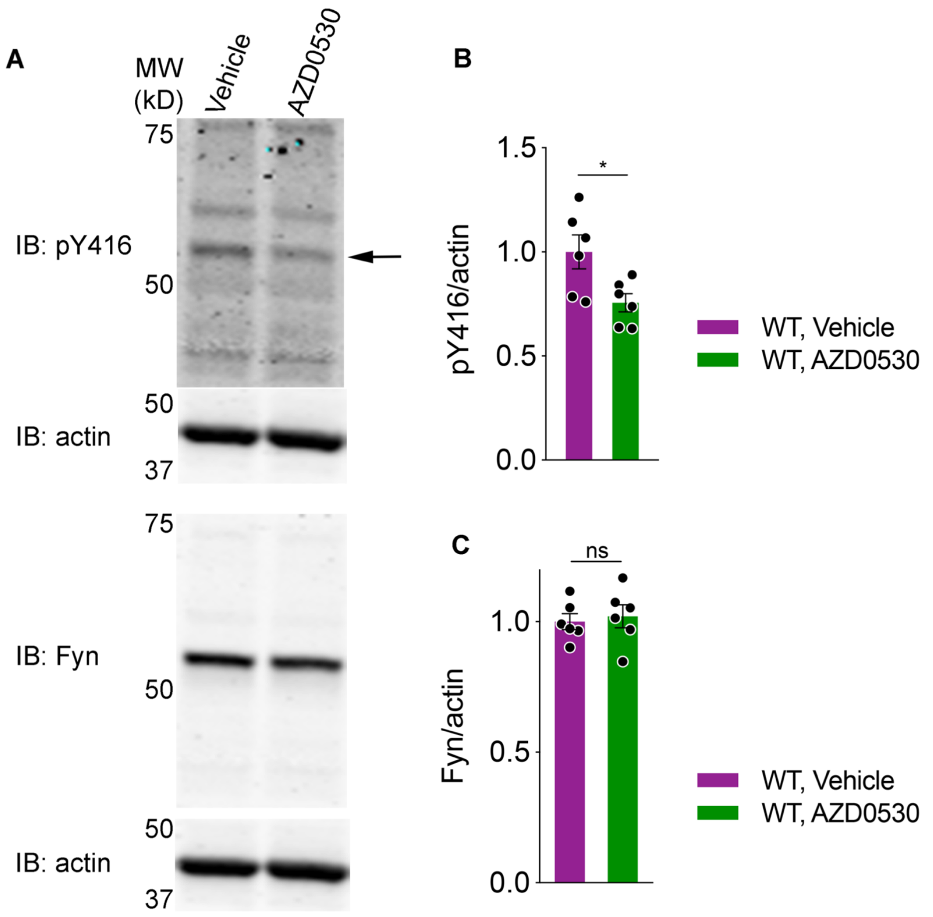

Supplementary Figure S1. Reduced Fyn Activation in Mice Fed AZD0530-Containing Food.
(A) Representative blots using anti-pY416, Fyn, and ß-actin antibodies of the RIPA-soluble fraction of the hippocampus of WT mice treated with Vehicle or AZD0530 for 9 months.
(B) Quantification of pY416-immunoreactive bands from the immunoblot from **A** by densitometric analysis. The bands indicated by an arrow in **A** were quantified. The band intensity was normalized to that of ß-actin and then normalized to the mean of the Vehicle-treated WT group. Data are represented as mean ± SEM. n = 6 /group. *$p < 0.05$; t-test.
(C) Quantification of Fyn-immunoreactive bands from the immunoblot in **A** by densitometric analysis. The band intensity was normalized to that of ß-actin and then normalized to the mean of the Vehicle-treated WT group. Data are represented as mean ± SEM. n = 6 /group. t-test.

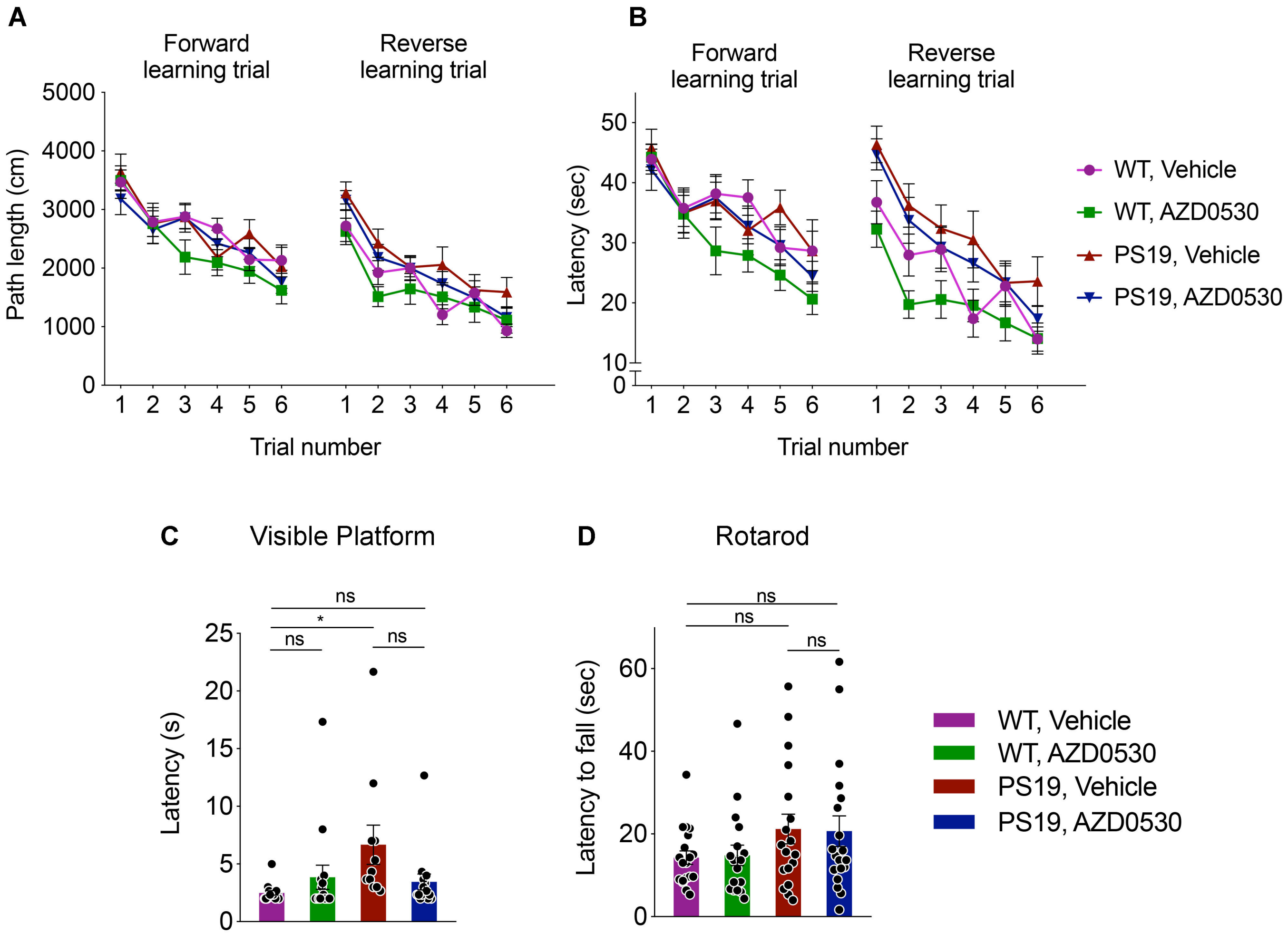

Supplementary Figure S2. Behavioral Tests of PS19 or WT Mice Treated with AZD0530 or Vehicle

(A) Morris water maze distance traveled for forward and reverse swims in 8-month-old PS19 and WT mice after 6 months of treatment. Pathlength is measured as the total distance traveled (in cm) before the mouse reaches the submerged platform. Data are mean ± SEM. n = 11-15 /group. One-way ANOVA.

(B) Morris water maze latency to target for forward and reverse swims in 8-month-old PS19 and WT mice after 6 months of treatment. The latency was measured as the time for the mouse to find a submerged platform in a forward and a reverse swim after a platform relocation. Data are mean ± SEM. n = 11-15 /group. One-way ANOVA.

(C) Morris water maze visible platform trial after reverse swim. Latency is measured as the average amount of time the mouse takes to reach the flagged platform in an average of 12 trials or until the latency has plateaued for 3 trials, whichever comes first. Data are mean ± SEM. n = 11-15 /group, each dot from one mouse. *$p < 0.05$, One-way ANOVA with Holm-Sidak's multiple comparisons test.

(D) Rotarod trials in 8-month-old PS19 and WT mice of the prophylactic cohort. Latency to fall is measured as the time it takes to fall from the rotating, accelerating rod. Each data represents the average of 5 trials for one mouse. Data are mean ± SEM. n = 18-19 /group. One-way ANOVA.

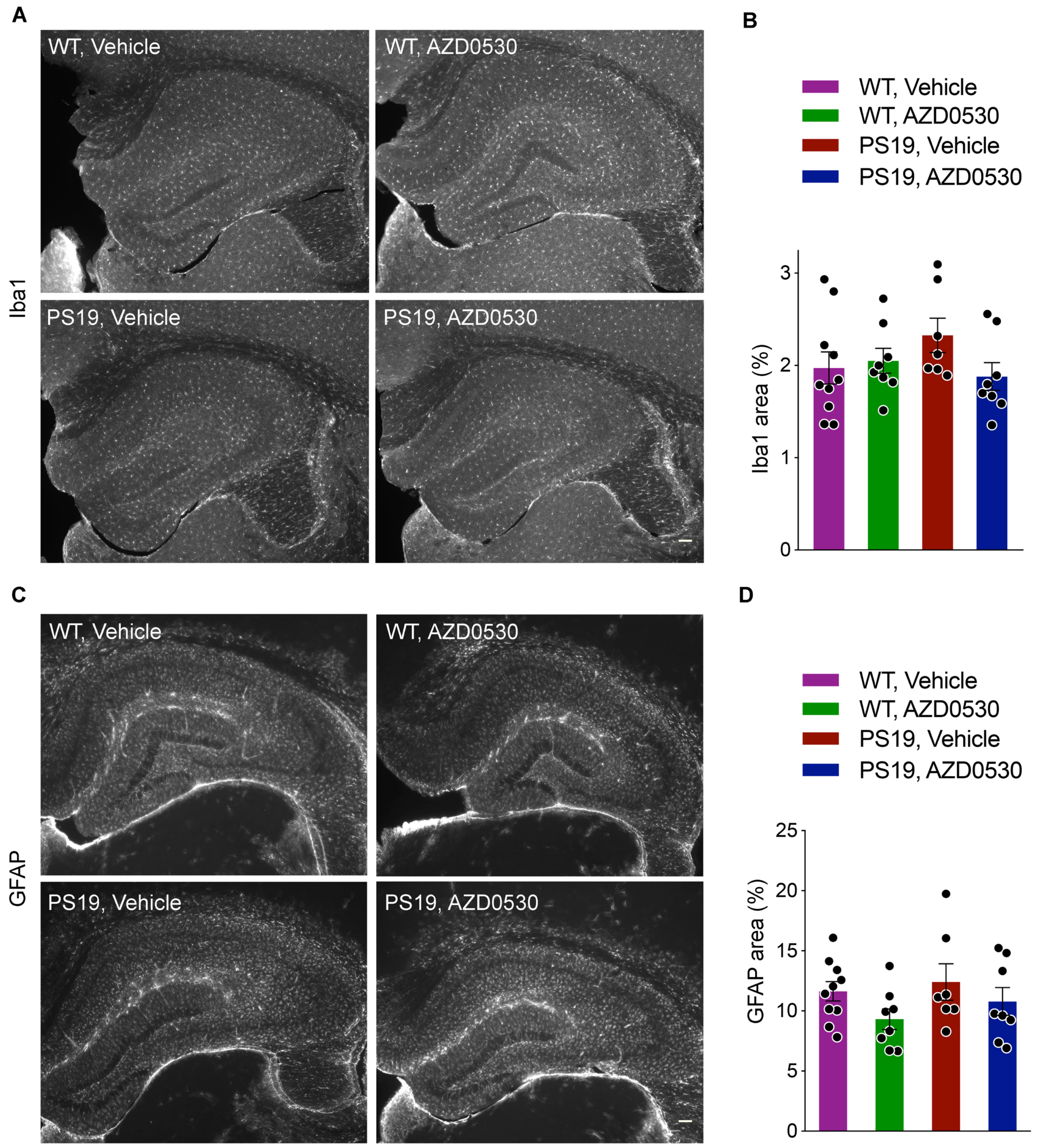

Supplementary Figure S3. Low Magnification Survey of Gliosis in PS19 Mice Unaffected by AZD0530.

(A) Representative images of Iba1 immunostaining in the hippocampus in 9-month-old PS19 and WT mice after 7 months of treatment. Scale bar, 100 μm.

(B) Quantification of Iba1-positive area (%) in the hippocampus in 9-month-old PS19 and WT mice collected after 7 months of treatment. Data are mean ± SEM. n = 7-10 /group. One-way ANOVA.

(C) Representative images of immunofluorescent staining GFAP in the hippocampus in 9-month-old PS19 and WT mice after 7 months of treatment. Scale bar, 100 μm.

(D) Quantification of GFAP-positive area (%) in the hippocampus in 9-month-old PS19 and WT mice

collected after 7 months of treatment. Data are mean ± SEM. n = 7-10 /group. One-way ANOVA.

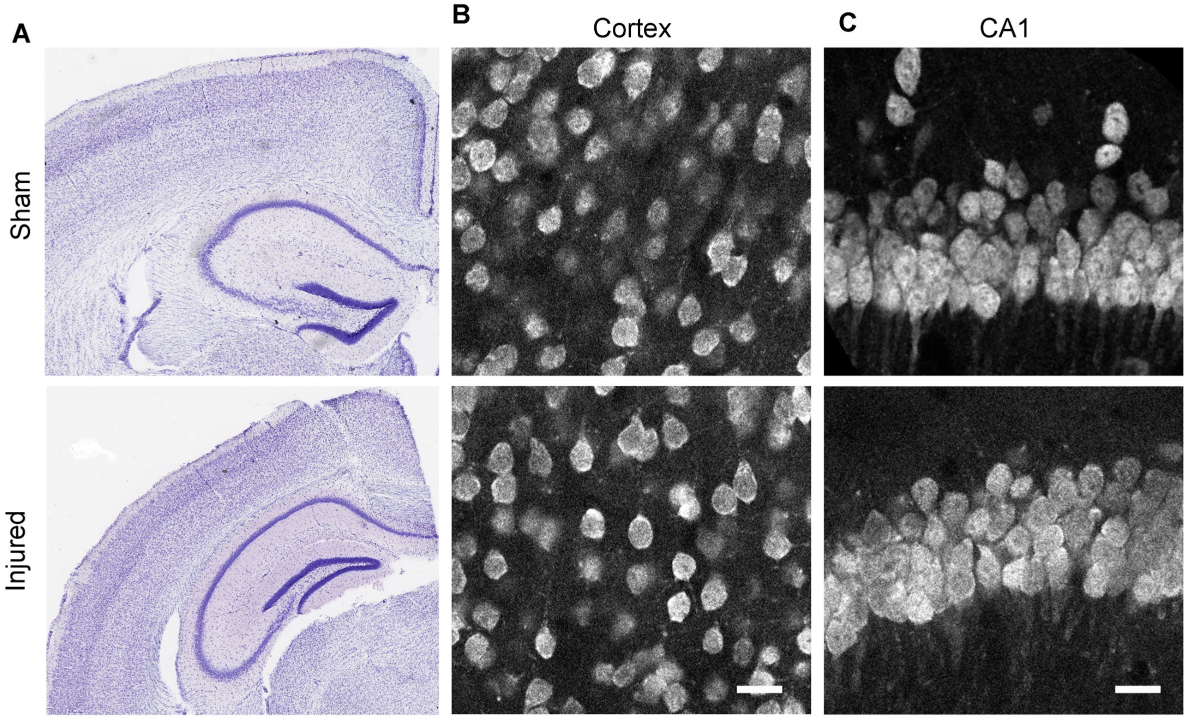

Supplementary Figure S4. Minimal Tissue Damage or Neuronal Loss after rmTBI/Stress.
(A) Representative cresyl violet stained images of Sham Vehicle-treated (Sham) and Injured Vehicle-treated (Injured) of the cortex and hippocampal regions containing the injury site collected more than 3 months after injury.
(B) Representative NeuN stained images of Sham Vehicle-treated (Sham) and Injured Vehicle-treated (Injured) coronal sections of cerebral cortex within 0.5-1 mm medial to the site of injury, using 20X magnification. Scale bar, 20 µm.
(C) Representative NeuN stained images of sham vehicle treated (Sham) group and injured vehicle treated (Injured) coronal sections from the CA1 region of the hippocampus within 1 mm of the site of injury, using 20X magnification. Scale bar, 20 µm.

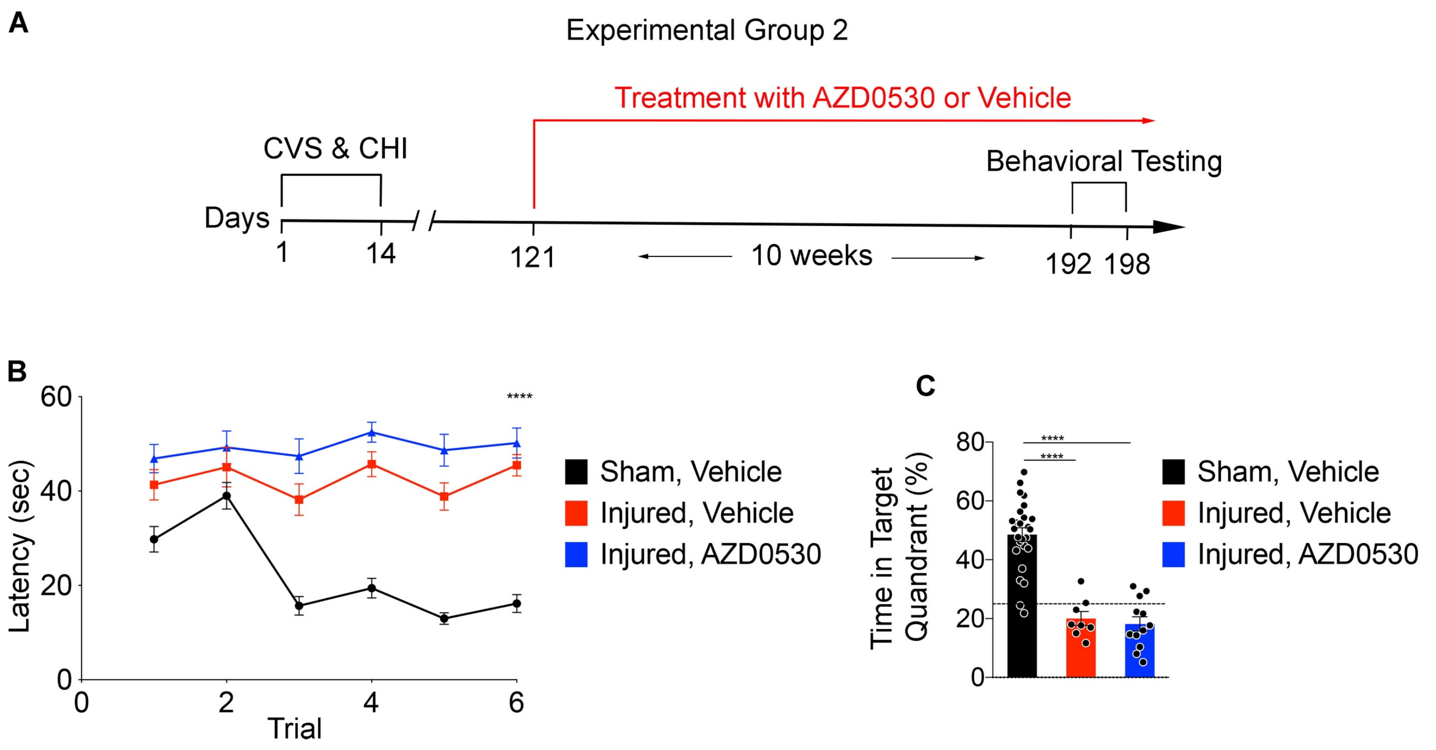

Supplementary Figure S5. Fyn Inhibitor Treatment of Chronic rmTBI/Stress Mice.

(A) Timeline for a second cohort of mice that underwent a similar 14 days of chronic variable stress (CVS) plus closed head injury (CHI) or Sham CVS & CHI paradigm, and then starting on Day 121 were treated with either AZD0530 (5 mg/kg/d) or Vehicle for 10 weeks. The mice subsequently underwent Morris water maze testing at 11 months of age.

(B) Latency to reach a hidden platform in reverse Morris water maze for 11-month-old WT mice from Sham Vehicle-treated (SV), Injured Vehicle-treated (IV), and Injured AZD0530-treated (IA) groups. Latency is measured as the time it takes for the mouse to reach the hidden platform. Both Injured groups exhibited longer latency to the hidden platform compared to the Sham group, but the two Injured groups were not significantly different from one another. Data are mean ± SEM. n = 8-26 /group. Two-way ANOVA, ****$p < 0.0001$; Tukey's multiple comparisons test.

(C) Morris water maze probe trial performed 24 hours after training trials in **B** for 11-month-old WT mice from SV, IV, and IA groups. Neither mice from IV nor IA groups demonstrated preference towards the target quadrant. Dashed line indicates random chance performance of 25% in the target quadrant. Data are mean ± SEM. n = 8-26 /group, each dot is one mouse. Two-tailed Wilcoxon signed rank test for non-Gaussian distribution versus random chance: SV, ***$p = 0.0001$; IV and IA, n.s., $p > 0.05$. One way ANOVA, Tukey's multiple comparisons test: SV vs IV, ****$p < 0.0001$; IV vs IA, n.s., $p > 0.05$; SV vs IA, ****$p < 0.0001$.

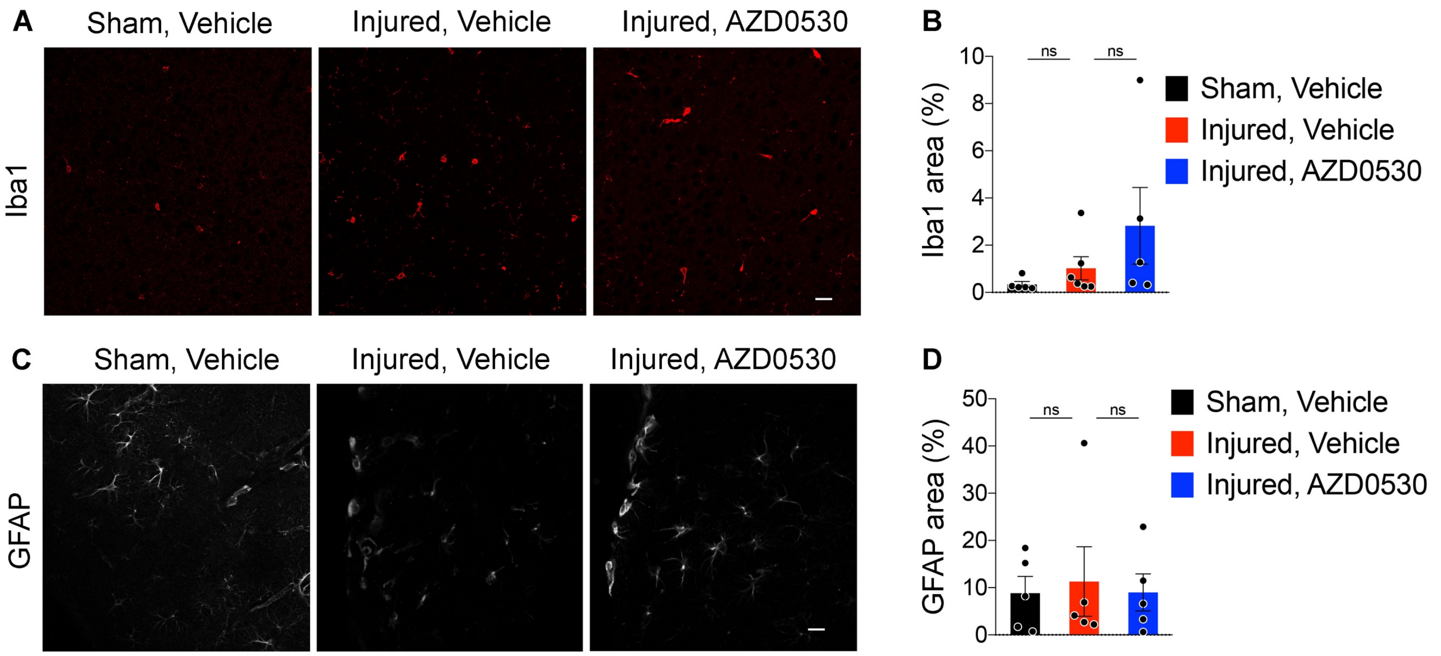

Supplementary Figure S6. Minimal Microgliosis and Astrogliosis Months after rmTBI/Stress Unaffected by Fyn Inhibitor.

(A) Representative images of immunofluorescent staining Iba1 of cortical sections in the same region as Fig 5F, 5H in 7.5-month-old WT mice from Sham Vehicle-treated (SV), Injured Vehicle-treated (IV), and Injured AZD0530-treated (IA) groups. Scale bar, 20 μm.

(B) Quantification of Iba1-positive area (%) in 7.5-month-old WT mice from SV, IV, and IA groups. Data are mean ± SEM. n = 5 / group, each dot is one mouse. One way ANOVA with Dunnett's multiple comparisons test,

(**C)** Representative images of immunofluorescent staining GFAP of cortical sections in the same region as in Fig. 5F, 5H in 7.5-month-old WT mice from SV, IV, and IA groups. Scale bar, 20 μm.

(D) Quantification of GFAP-positive area (%) in 7.5-month-old WT mice from SV, IV, and IA groups. Data are mean ± SEM. n = 5 / group, each dot is one mouse. One way ANOVA with Tukey's multiple comparison test.

Supplementary Table S1. Mouse Cohorts

Exp	Genotype, Treatment	N (M, F)	Genetic Back-ground	Drug Dosing	Avg Age at Behavior Tests	Avg Age at Sacrifice	Avg Weight at Sacrifice	Experiments Conducted
1	WT, Vehicle<br>WT, AZD0530<br>PS19, Vehicle<br>PS19, AZD0530	(8,3)<br>(7,2)<br>(5,5)<br>(5,5)	B6C3<br>B6C3<br>B6C3<br>B6C3	5 mg/kg/d*	8 months	9 months	39.5 g<br>43.5 g<br>32.3 g<br>36.2 g	-Morris Water Maze (combined with Cohort 2)<br>-Passive Avoidance Test (combined with Cohort 2)<br>-Immunohistochemistry: AT8, PHF1, CD68/Iba1, GFAP, SV2A, pY18
2	WT, Vehicle<br>WT, AZD0530<br>PS19, Vehicle<br>PS19, AZD0530	(4,5)<br>(6,3)<br>(3,6)<br>(2,7)	B6C3<br>B6C3<br>B6C3<br>B6C3	5 mg/kg/d*	8 months	11 months	42.0 g<br>43.0 g<br>32.8 g<br>32.2 g	-Morris Water Maze (combined with Cohort 1)<br>-Passive Avoidance Test (combined with Cohort 1)<br>-Biochemistry: Fyn, pY416
3	WT, Sham, Vehicle<br>WT, CVS&CHI, Vehicle<br>WT, CVS &CHI, AZD0530	(14,0)<br>(13,0)<br>(15,0)	C57BL6J<br>C57BL6J<br>C57BL6J	5 mg/kg/d#	7 months	7.5 months	25.3 g<br>25.8 g<br>22.7 g	-Rotarod Test<br>-Morris Water Maze<br>-Novel Object Recognition<br>-Immunohistochemistry: Tau, PHF1, GFAP, Iba-1
4	WT, Sham, Vehicle<br>WT, CVS&CHI, Vehicle<br>WT, CVS&CHI, AZD0530	(26,0)<br>(8,0)<br>(12,0)	C57BL6J<br>C57BL6J<br>C57BL6J	5 mg/kg/d#	11 months	12 months	35.7 g<br>34.3 g<br>35.3 g	-Morris Water Maze<br>-Cresyl Violet, NeuN

* With food pellets
# Oral gavage, divided dosage at 7 am and 7 pm
